# Dental Plaque Microbial Resistomes of Periodontal Health and Disease and Their Changes after Scaling and Root Planing Therapy

**DOI:** 10.1128/mSphere.00162-21

**Published:** 2021-07-21

**Authors:** Yutong Kang, Bianjin Sun, Yiju Chen, Yongliang Lou, Meiqin Zheng, Zhenjun Li

**Affiliations:** a State Key Laboratory for Infectious Disease Prevention and Control, National Institute for Communicable Disease Control and Prevention, Chinese Center for Disease Control and Prevention, Beijing, China; b Eye Hospital and School of Ophthalmology and Optometry, Wenzhou Medical University, Wenzhou, Zhejiang, China; c National Clinical Research Center for Ocular Diseases, Wenzhou, Zhejiang, China; d Wenzhou Key Laboratory of Sanitary Microbiology, Key Laboratory of Laboratory Medicine, Ministry of Education, School of Laboratory Medicine and Life Sciences, Wenzhou Medical University, Wenzhou, Zhejiang, China; University of Nebraska Medical Center

**Keywords:** periodontitis, metagenomic analysis, microbial community, antibiotic resistance genes, metal resistance genes, mobile genetic elements

## Abstract

The human oral microbial community has been considered a reservoir of antibiotic resistance. Currently, the effects of periodontitis and the scaling and root planing (SRP) treatment on the performance of antibiotic-resistant genes (ARGs) and metal-resistant genes (MRGs) in the dental plaque microbiota are not well characterized. To explore this issue, we selected 48 healthy-state (HS), 40 periodontitis-state (PS; before treatment), and 24 resolved-state (RS; after SRP treatment) metagenomic data of dental plaque samples from the Sequence Read Archive (SRA) database. NetShift analysis identified Fretibacterium fastidiosum, Tannerella forsythia, and Campylobacter rectus as key drivers during dental plaque microbiota alteration in the progression of periodontitis. Periodontitis and SRP treatment resulted in an increase in the number of ARGs and MRGs in dental plaque and significantly altered the composition of ARG and MRG profiles. Bacitracin, beta-lactam, macrolide-lincosamide-streptogramin (MLS), tetracycline, and multidrug resistance genes were the main classes of ARGs with high relative abundance, whereas multimetal, iron, chromium, and copper resistance genes were the primary types of MRGs in dental plaque microbiota. The cooccurrence of ARGs, MRGs, and mobile genetic elements (MGEs) indicated that a coselection phenomenon exists in the resistomes of dental plaque microbiota. Overall, our data provide new insights into the standing of the distribution of ARGs and MRGs in oral microbiota of periodontitis patients, and it was possible to contribute to the understanding of the complicated correlations among microorganisms, resistomes, and MGEs.

**IMPORTANCE** The emergence and development of resistance to antibiotics in periodontal pathogens have affected the success rate of treatment for periodontitis. The development of new antibacterial strategies is urgently needed to help control and treat periodontal disease, and dental plaque microbiome studies offer a promising new angle of attack. In this study, we investigated the dental plaque microbiota and resistomes in periodontal health and disease states and their changes after SRP therapy. This is the first analysis of the profile of the microbial community and antibiotic and metal resistance genes in dental plaque by the metagenomic approach, to the best of our knowledge. Monitoring the profile of these resistomes has huge potential to provide reference levels for proper antibiotics use and the development of new antimicrobial strategies in periodontitis therapy and thereby improve actual efficacy of the treatment regimens.

## INTRODUCTION

Periodontitis is a chronic infectious disease related to changes in the subgingival microbiome of individual tooth sites that can destroy the structure that supports teeth (1, 2). Clinical treatment of periodontitis is currently difficult considering its high recurrence rate (3). Antibiotics are considered an invaluable resource to manage orofacial infections (4). Past research has suggested that adjunctive application of systemic antibiotics in patients with chronic periodontitis have better clinical effects than no antibiotics (5–8). When the subgingival microbial profiles are unknown, however, most periodontal antibiotic therapeutic regimens are prescribed based on the experience of the treating physician (9). In that case, if periodontal pathogens are intrinsically resistant or poorly susceptible to the selected antibiotic drug, failure of clinical antimicrobial treatment is inevitable (10). Of concern, empirical antibiotic prescribing has resulted in the development of a wide range of microbial resistances, making commonly used antibiotics ineffective (11).

With the emergence and development of antibiotic resistance in oral pathogens, metal and metal oxide nanoparticles (NPs) have received considerable attention in dentistry given their good antibacterial, antiadhesive, and delivery capabilities (12). It has been documented that NPs, such as zinc oxide, silver, copper oxide, nickel, nickel oxide, tungsten trioxide, gold, and titanium dioxide (TiO2), exhibit antibacterial activity against oral bacteria (13–19). With respect to nanoparticulate metals, most attention has been paid to the antimicrobial properties of silver and copper (20, 21). A recent study, however, showed a high frequency of silver-resistant genes in endodontic bacteria (22). Notably, over the past few decades, the coselection phenomenon of ARGs and MRGs frequently has been observed in a variety of environments (23, 24). Heavy-metal pollution in the natural environment can play a significant role in maintaining and enhancing antibiotic resistance (25–28). Compared with less human-associated bacteria, the signatures of cooccurrence between ARGs and MRGs are much more frequent in human pathogens (28). A comprehensive understanding of the profiles of ARGs and MRGs in the oral microbiome, therefore, is crucial for the development of new, effective, and economic alternative antimicrobial agents in the future.

Previous studies have evaluated the composition and changes of ARGs and MRGs in the dental plaque mostly through traditional culture- and amplification-based genetic methods (22, 29–31). These methods, however, did not consider unculturable bacteria, the limited number of available primers for targeted genes, amplification bias, or low throughput. Because of efficiency and primer independence, the metagenomic shotgun sequencing has been used widely in ARG investigation and comparison (32, 33). Metagenomic shotgun sequencing has led to a paradigm shift in our understanding of antibiotic resistance, which now places the primary focus on a broader concept of an oral resistome rather than on the carriage of antibiotic resistance in cultivable pathogens (34). The collection of all resistance genes in both pathogens and commensal microorganisms is referred to as the resistome (35).

Here, we performed a metagenomic analyses on 112 dental plaque samples obtained from the National Centre for Biotechnology Information (NCBI) SRA database. To the best of our knowledge, this is the first study based on metagenomic sequencing to report the distribution patterns of ARGs and MRGs of the dental plaque microbiota in HS, PS (before treatment), and RS (after treatment). Subsequently, we characterized the correlation between the microbial communities and resistomes (ARGs and MRGs) and the roles of the microbial communities and MGEs in changes in these resistomes, and, finally, revealed the cooccurrence of ARGs, MRGs, and MGEs in the dental plaque microbiome. This study aimed to investigate the changes and profiles of taxonomic classification and resistance genes in the oral microbiome of patients with periodontitis. This work provides an important reference for the selection of antibiotics therapy as well as the development of effective metal or metal oxide nanomaterials. This is significant to ensure effective and safe treatment and to avoid the increase in microbial resistance in dental practice.

## RESULTS

### Microbial community in dental plaque among HS group, PS group, and RS group alpha diversity and beta diversity of microbial community in dental plaque.

We obtained a total of 1,529,804,857 high-quality nonhuman sequences with an average of 13,658,972 sequences per sample and used the sequences for further analysis. A total number of 243 bacterial, 14 viral, and 1 fungal species were identified by sequence analysis using MetaPhlAn software. Thirty bacterial species with the highest abundance are shown in Fig. S1a in the supplemental material. The sum of their average relative abundances was 64.87%, 56.65%, and 57.82% for HS, PS, and RS groups, respectively. Figure S1b shows the distribution and relative abundance of all virus and fungal species.

10.1128/mSphere.00162-21.1 FIG S1 (a) Heatmap and marginal histogram reporting the abundance and positive rate of the 30 most abundant species. (b) Heatmap of virus and fungal relative abundance at the species level. (c) Boxplots of the inverse Simpson indexes of the microbial community at the species level in HS group, PS group, and RS group. (d) PCoA of Jaccard distance between groups. Download FIG S1, PDF file, 0.7 MB. Copyright © 2021 Kang et al. 2021 Kang et al. https://creativecommons.org/licenses/by/4.0/ This content is distributed under the terms of the Creative Commons Attribution 4.0 International license.

**FIG 1:**
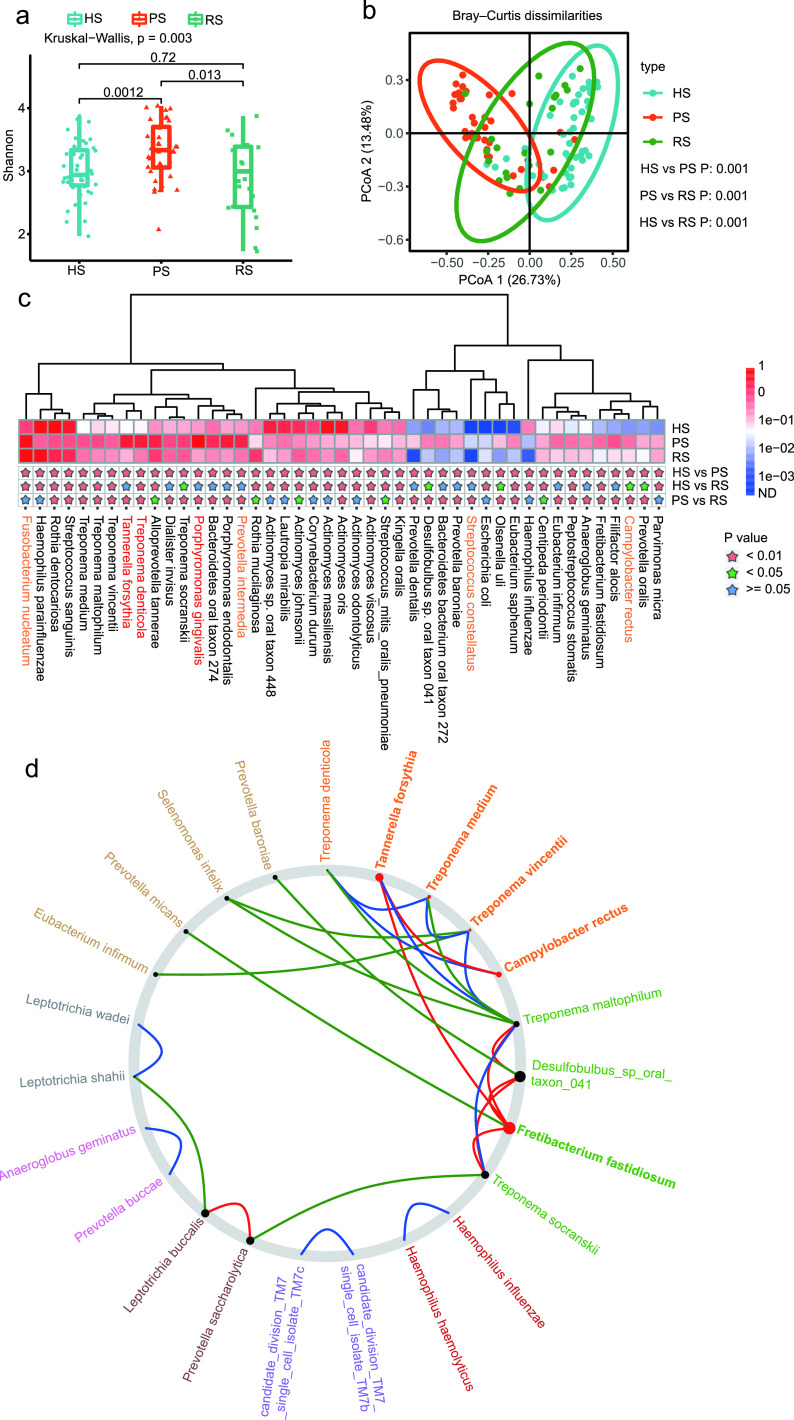
Microbial community in the dental plaque among the HS group, PS group, and RS group. (a) Box plots of the Shannon-Wiener indexes of the microbial community at the species level in the HS group, PS group, and RS group. (b) PCoA of Bray-Curtis distance between the groups. (c) Relative abundance of the top 45 most different species across the groups. Species at a *P* value of <0.01 are marked with a pink star, a *P* value of <0.05 with a green star, and a *P* value of ≥0.05 with a blue star. The red typeface denotes the three members of the red complex; the orange typeface denotes the four members of the red complex. (d) We treated the HS and RS (after treatment) as the control and individuals with the PS (before treatment) as the case. Driver species are represented by bigger red nodes with a higher NESH score. Edge (line) is assigned between the nodes; green edges, association present only in the control; red edges, association present only in the case; and blue, association present in both control and case.

The Shannon and the Simpson indices in the PS group were significantly higher than those in the HS group and RS group. We did not observe a statistically significant difference between the HS and RS group (Fig. 1a and Fig. S1c). We identified significant differences in community structure (Bray-Curtis) and composition (Jaccard) between any two of the three groups at the species level (Fig. 1b and Fig. S1d).

### Identification of differentially abundant microbial signatures.

Figure 1c shows the top 45 most different species across the groups. Note that 13 of the species showed significant enrichment in the PS group compared with HS and RS groups, but significant differences did not exist between the HS group and RS group. These species, including Tannerella forsythia, Dialister invisus, Porphyromonas gingivalis, *Bacteroidetes* oral taxon 274, Porphyromonas endodontalis, Prevotella intermedia, Prevotella dentalis, *Bacteroidetes* bacterium oral taxon 272, Prevotella baroniae, Streptococcus constellatus, Eubacterium infirmum, Anaeroglobus geminatus, and Filifactor alocis, were considered therapeutic targets for periodontitis when using antibiotics as an adjunct to SRP.

### Identification of driver species based on NetShift analysis.

To gain insight into the role of important species in the development of periodontitis, we identified driver species in the microbiome network between the case and control based on the NetShift method. We treated individuals with HS and RS (after treatment) as the controls and individuals with the PS (before treatment) as the cases. Comparison of control and case networks revealed Fretibacterium fastidiosum, Tannerella forsythia, and Campylobacter rectus as the driver nodes (species) with higher neighbor shift (NESH) scores (red color and bigger nodes), which significantly affected the overall community structure of the oral microbiome (Fig. 1d).

### Antibiotic-resistant genes in dental plaque among the HS group, PS group, and RS group. (i) Broad-spectrum profile of ARG abundances in various groups.

**FIG 2:**
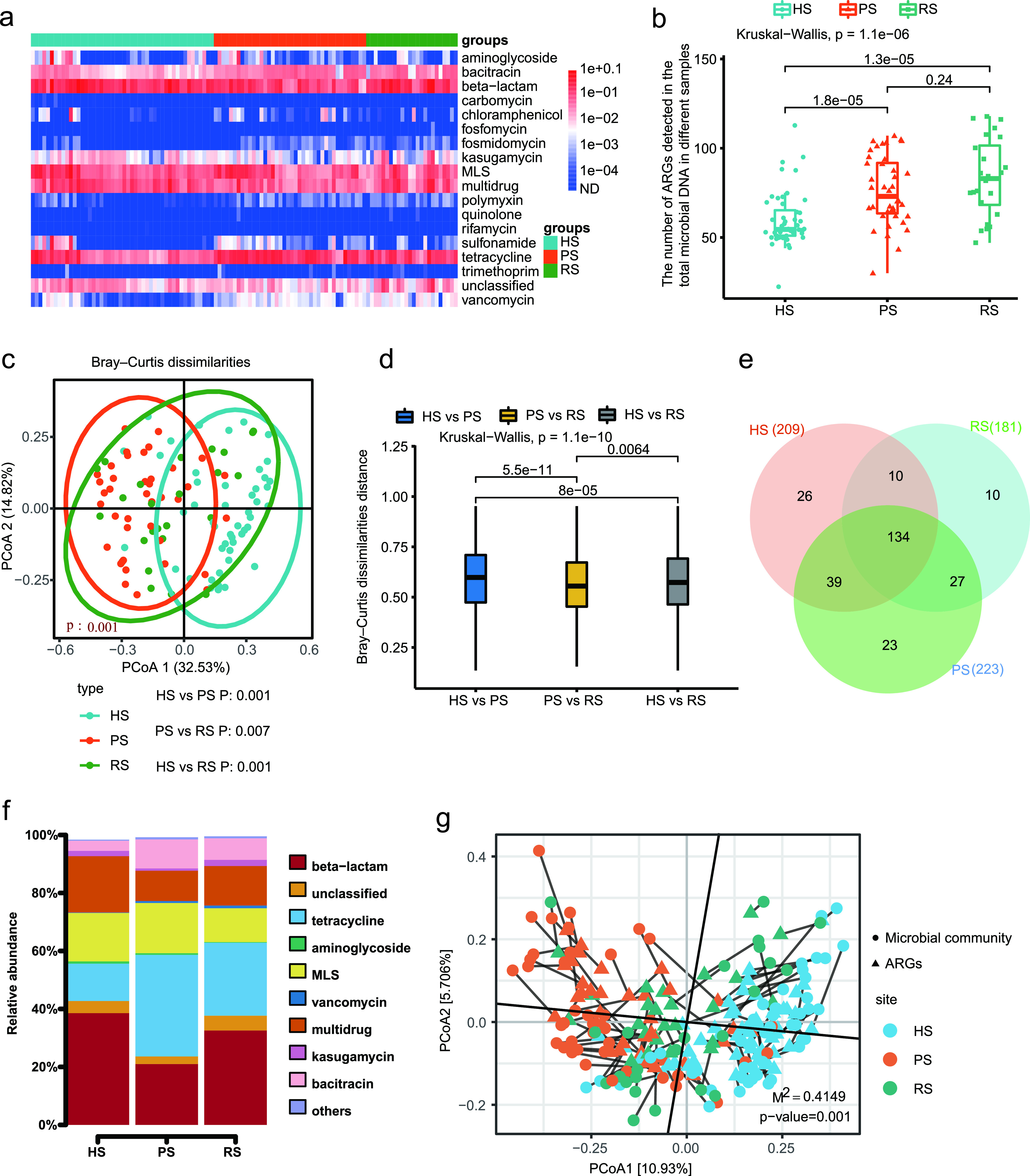
Antibiotic resistome differences among the HS group, PS group, and RS group. (a) Broad-spectrum quantitative profile of the ARG types (copy/cell) in 112 dental plaque samples. (b) Box plots of the number of ARG subtypes in HS group, PS group, and RS group. (c) PCoA of Bray-Curtis distance between groups. (d) Box plots showing Bray-Curtis distance between sample types. (e) Venn diagram depicting the number of shared and unique ARG subtypes among HS group, PS group, and RS group. (f) The relative abundance (%) of shared ARGs. (g) Procrustes analysis of the correlations between ARGs and microbial community structure based on Bray-Curtis dissimilarity matrix.

We investigated ARGs across a broader spectrum without PCR, based on a comprehensive reference resistance gene sequence database consisting of 1,244 ARG subtypes of 24 ARG types. In total, we detected 18 ARG types and 269 ARG subtypes in the dental plaque microbiota of the study subjects. The top six ARG types that had a higher relative abundance and higher positivity rate, which we considered the representative ARGs, were beta-lactam, tetracycline, multidrugs, macrolide-lincosamide-streptogramin, bacitracin, and kasugamycin. The mean relative abundance of carbomycin, fosfomycin, quinolone, or rifamycin was 10^−7^ to 10^−6^ copies per cell, which was markedly lower than that for other ARGs (Fig. 2a and Fig. S2a). The top 30 ARG subtypes are shown in Fig. S2b, including common resistance genes such as *ermB*, *tet*(32), *tetQ*, *tetW*, *tetM*, and *cfxA*.

10.1128/mSphere.00162-21.2 FIG S2 (a) Comparison of different ARG abundances at the type level in the 112 dental plaque samples. (b) Heatmap and marginal histogram reporting the abundance and positive rate of the 30 most abundant ARG subtypes. (c) PCoA (Jaccard distance) plot showing the differences of ARG composition among different groups. (d) Boxplots showing Jaccard distance between-sample type. Download FIG S2, PDF file, 0.6 MB. Copyright © 2021 Kang et al. 2021 Kang et al. https://creativecommons.org/licenses/by/4.0/ This content is distributed under the terms of the Creative Commons Attribution 4.0 International license.

### (ii) Shared ARGs among the HS group, PS group, and RS group.

**FIG 3:**
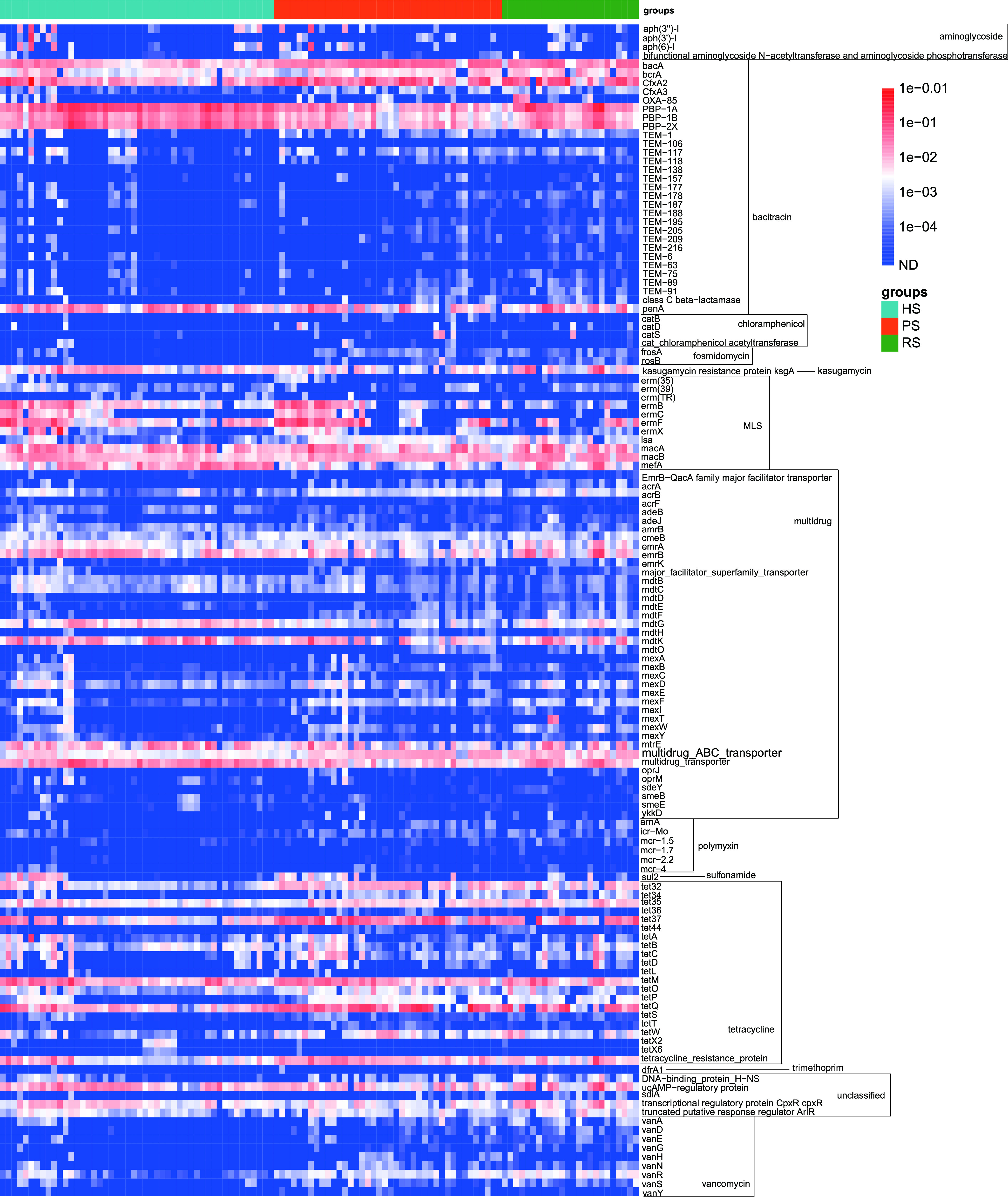
Abundance of the 134 shared ARGs by the 112 samples (copy/cell).

Among the 269 detected subtypes, the profile of 134 shared ARG subtypes of the three groups is shown in Fig. 3. A total of 134 ARG subtypes belonging to 14 types were shared by all study subjects. According to a Venn diagram analysis, we found 209 ARG subtypes in the HS group and identified 223 and 181 ARG subtypes in the PS and RS groups, respectively (Fig. 2e). The shared ARGs accounted for 98.39% ± 5.85%, 99.23% ± 0.91%, and 99.55% ± 0.57% of the total abundance of ARGs detected in HS, PS, and RS groups, respectively. These shared ARG subtypes, with the proportion closer to 100%, likely represent stably present ARG subtypes in dental plaque microbiota, while periodontal inflammatory condition and SRP treatment merely cause fluctuations in their abundance (Fig. 2f).

### (iii) Antibiotic resistome differences among the HS group, PS group, and RS group.

There was no significant difference in the number of ARG subtypes between the PS group and RS group, but the numbers of ARG subtypes in the PS group and RS group were significantly greater than that in the healthy group (Fig. 2b). Principal coordinate analysis (PCoA) based on Bray-Curtis distance and Jaccard distance showed that the comprehensive composition of the antibiotic resistome of the three groups was very different, with a clear separation of each experimental group (Fig. 2c and Fig. S2c). Note that although the SRP treatment could significantly change the composition of ARGs, it had little effect on the number of ARGs. Grouped comparisons of pairs of Bray-Curtis dissimilarity and Jaccard index values demonstrated that the similarity of the HS versus the RS group was significantly higher than the similarity of the HS versus the PS group (Fig. 2d and Fig. S2d). Although there was a significant difference in ARG composition between the RS group and HS group, the extent of the difference between the two groups was significantly reduced compared with the extent of the difference between the PS group and HS group.

### (iv) Correlation network of cooccurring ARG subtypes and microbial taxa.

The Procrustes tests depicted overall correlations between ARGs and microbial communities based on the Bray-Curtis dissimilarity matrix (Procrustes sum of squares, M^2^ = 0.4149, *r* = 0.7649; significance, 0.001; permutation, free; number of permutations, 999) (Fig. 2g). The ARG profile showed overall significant correlations with microbial communities. The Procrustes analysis suggested that microbial community composition shapes ARG distribution in the dental plaque microbiota.

We applied the network analysis to explore detailed cooccurrences between the specific ARG subtypes and microbial taxa. The potential hosts of ARGs could be tracked by way of nonrandom cooccurrence patterns (32, 36, 37). The correlation network was built based on the significant correlations (Spearman’s ρ, >0.6; Q value, <0.01) between ARG subtypes and microbial taxa that occurred in at least 56 of the 112 total samples. The detailed cooccurrence patterns between species and ARG subtypes are listed in Table S2. Figure S3 shows 57 nodes (32 microbial taxa and 25 ARG subtypes) and 85 edges. We proposed that 32 species contained 25 ARG subtypes conferring resistance to six kinds of antibiotics (multidrugs, tetracycline, unclassified, MLS, beta-lactam, and kasugamycin).

10.1128/mSphere.00162-21.3 FIG S3 Network analysis showing the cooccurrence pattern between ARGs and microbial community compositions based on Spearman correlation analysis. A connection represents a strong (Spearman’s *r*, >0.6) and significant (*P*  < 0.01) correlation. The nodes were colored according to species and ARG types. The size of each node is proportional to the number of connections. Download FIG S3, PDF file, 0.4 MB. Copyright © 2021 Kang et al. 2021 Kang et al. https://creativecommons.org/licenses/by/4.0/ This content is distributed under the terms of the Creative Commons Attribution 4.0 International license.

10.1128/mSphere.00162-21.9 TABLE S2 Potential ARG hosts information revealed by cooccurrence between ARG subtypes and microbial taxa. Download Table S2, DOCX file, 0.02 MB. Copyright © 2021 Kang et al. 2021 Kang et al. https://creativecommons.org/licenses/by/4.0/ This content is distributed under the terms of the Creative Commons Attribution 4.0 International license.

Among 32 species, Haemophilus parainfluenzae was the potential host of the majority of ARGs, including beta-lactam (PBP-1A, PBP-1B, and PBP-2X), kasugamycin (kasugamycin resistance protein KsgA), MLS (*mefA*), multidrug (*emrA*, *emrB*, *mdtG*, multidrug transporter), and unclassified (cyclic AMP-regulatory protein, transcriptional regulatory protein CpxR) resistance genes. Some species (Streptococcus sanguinis, Streptococcus mitis oralis pneumoniae, Treponema medium, Treponema maltophilum, Lautropia mirabilis, and Granulicatella adiacens) were also predicted to harbor four or more ARG subtypes. Other bacterial species were associated with three or fewer ARG subtypes. For instance, Tannerella forsythia carried two ARG subtypes to bacitracin (*bcrA*) and tetracycline [*tet*(37)]. Treponema denticola was a potential host for three ARG subtypes, two tetracycline-resistant genes [*tet*(32) and *tetW*] and a bacitracin-resistant gene (*bacA*). A multidrug-resistant gene (i.e., multidrug ABC transporter) could be contained in Fusobacterium nucleatum. Prevotella nigrescens was associated with the tetracycline-resistant gene [*tet*(37)].

### Metal-resistant genes in dental plaque among the HS group, PS group, and RS group. (i) Broad-spectrum profile of MRG abundances in various groups.

**FIG 4:**
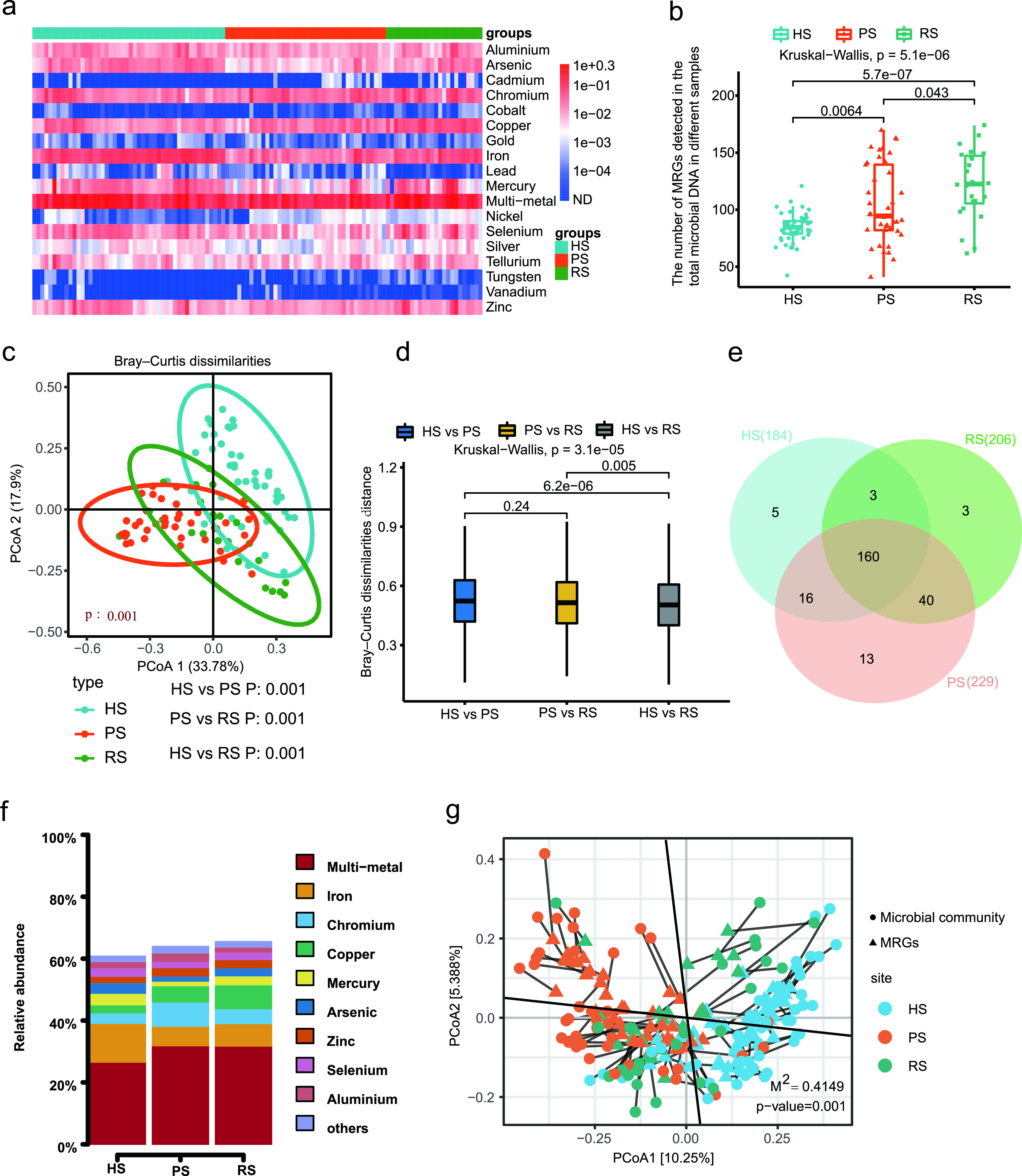
Metal resistome differences among HS group, PS group, and RS group. (a) Broad-spectrum quantitative profile of the MRG types (copies/cell) in 112 dental plaque samples. (b) Box plot comparing the number of MRG subtypes among the HS group, PS group, and RS group. (c) PCoA plot showing the similarities of the MRG subtype composition among the 112 dental plaque samples in three groups. (d) Box plots showing Bray-Curtis distance between sample types. (e) Venn diagram depicting the number of shared and unique MRG subtypes among the HS group, PS group, and RS group. (f) The relative abundance (%) of shared MRGs. (g) Procrustes analysis of the correlation between ARGs and microbial community based on the PCoA (Bray-Curtis) results of MRG subtype abundances and microbial communities abundances.

We detected 18 MRG types, including 240 subtypes, in the dental plaque microbiota of the study subjects. The relative abundance of multimetal resistance genes in the dental plaque was the highest, with a range from 0.060 to 1.586 copies/cell. Iron resistance genes were found with the second-highest relative abundance (range, 0.008 to 0.178 copies/cell), followed by chromium (range, 0.002 to 0.139 copies/cell) and copper (range, 0.004 to 0.134 copies/cell) resistance genes. The relative abundance of cobalt resistance genes in the dental plaque was the lowest, with a range from 0 to 8.39e−4 copies/cell (Fig. 4a and Fig. S4a). The top 30 MRG subtypes are shown in Fig. S4b.

10.1128/mSphere.00162-21.4 FIG S4 (a) Comparison of different MRG abundances at the type level in the 112 dental plaque samples. (b) Heatmap and marginal histogram reporting the abundance and positive rate of the 30 most abundant MRG subtypes. (c) PCoA (Jaccard distance) plot showing the differences of MRGs composition among different groups. (d) Boxplots showing Jaccard distance between-sample type. Download FIG S4, PDF file, 0.5 MB. Copyright © 2021 Kang et al. 2021 Kang et al. https://creativecommons.org/licenses/by/4.0/ This content is distributed under the terms of the Creative Commons Attribution 4.0 International license.

### (ii) Shared MRGs among the HS group, PS group, and RS group.

**FIG 5:**
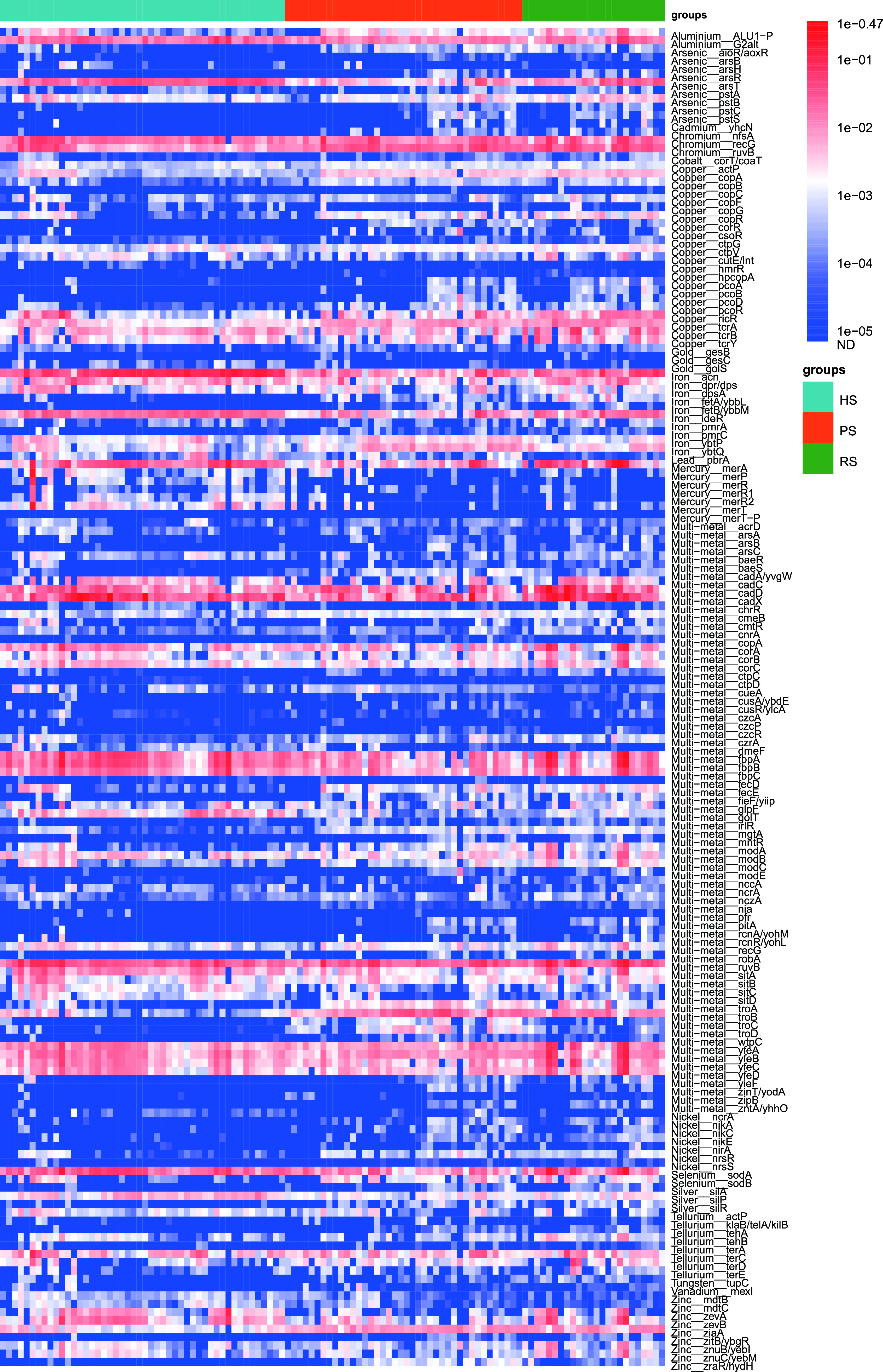
Abundance of the 160 shared MRGs by the 112 samples (copy/cell).

Among the 240 detected subtypes, the profile of 160 shared MRG subtypes of three groups is shown in Fig. 5. A total of 160 MRG subtypes belonging to 18 types were shared by all of the study subjects. Venn diagram analysis revealed that 184 MRG subtypes were found in the HS group, whereas 229 and 206 MRG subtypes were identified in the PS and RS groups, respectively (Fig. 4f). The shared ARGs accounted for 61.13% ± 9.58%, 64.30% ± 10.34%, and 65.86% ± 11.60% of the total abundance of MRGs detected in HS, PS, and RS groups, respectively (Fig. 4g). The proportion of shared MRGs among the three groups was markedly lower than the proportion of shared ARGs.

### (iii) Metal resistome differences among the HS group, PS group, and RS group.

The numbers of MRG subtypes in the PS and RS groups were also significantly higher than those in the HS group (Fig. 4b). PCoA based on Bray-Curtis distance and Jaccard distance showed that the comprehensive composition of metal resistome of the three groups was significantly different (Fig. 4c and Fig. S4c). Pairwise comparisons of Bray-Curtis dissimilarity and Jaccard index values demonstrated that the similarity between healthy and RS groups was significantly higher than the similarity between HS and PS groups (Fig. 4d and Fig. S4d). Similarly, although there were significant differences in the MRG composition between the RS group and the HS group, the degree of difference between the two groups was significantly reduced compared with the extent of the difference between the PS group and HS group.

### (iv) Correlation network of cooccurring MRG subtypes and microbial taxa.

The Procrustes tests depicted overall correlations between MRGs and microbial communities based on the Bray-Curtis dissimilarity matrix (Procrustes sum of squares, M^2^ = 0.396, *r* = 0.7772; significance, 0.001; permutation, free; number of permutations, 999) (Fig. 4g). The MRG profile had overall significant correlations with the microbial communities. Similarly, the Procrustes analysis suggested that microbial community composition might shape the distribution of MRG in the dental plaque microbiota.

We applied the network analysis to decipher the cooccurrence patterns between MRG subtypes and microbial taxa with high resolution. Figure S5 depicts 75 nodes (35 microbial taxa and 40 MRG subtypes) and 120 edges. The detailed cooccurrence between species and MRG subtypes is listed in Table S3. We proposed that 35 species contained 40 MRG subtypes conferring resistance to nine kinds of metals (arsenic, copper, iron, mercury, multimetal, selenium, silver, tellurium, and zinc). Among 35 species, Haemophilus parainfluenzae was the potential host of the most ARGs, including copper (*cutE-lnt*), mercury (*merA*), multimetal (*corA*, *corC*, *fbpA*, *fbpB*, *fbpC*, *golT*, *sitA*, *sitC*, *sitD*, *yfeC*, *yfeD*), selenium (*sodA*), and zinc (*zevA*, *zevB*, *znuB-yebI*, *znuC-yebM*) resistance genes. Some species (Lautropia mirabilis, Streptococcus sanguinis, Treponema socranskii, Treponema medium, Treponema maltophilum, Rothia dentocariosa, and Actinomyces oris) were predicted to harbor five or more MRG subtypes. Other bacterial species were associated with four or fewer MRG subtypes. For instance, Tannerella forsythia carried three MRG subtypes of multimetal-resistant genes (*cadA-yvgW*, *troB*, and *troD*). Treponema denticola was a potential host for three MRG subtypes of multimetal-resistant genes (*cadA-yvgW*, *troB*, and *troD*). Multimetal-resistant genes (*troB* and *troD*) also may be contained in Fusobacterium nucleatum.

10.1128/mSphere.00162-21.5 FIG S5 Network analysis showing the cooccurrence pattern between MRGs and microbial community compositions based on Spearman correlation analysis. A connection represents a strong (Spearman’s *r*, >0.6) and significant (*P*  < 0.01) correlation. The nodes were colored according to species and MRG types. The size of each node is proportional to the number of connections. Download FIG S5, PDF file, 0.4 MB. Copyright © 2021 Kang et al. 2021 Kang et al. https://creativecommons.org/licenses/by/4.0/ This content is distributed under the terms of the Creative Commons Attribution 4.0 International license.

10.1128/mSphere.00162-21.10 TABLE S3 Potential MRG hosts information revealed by cooccurrence between MRG subtypes and microbial taxa. Download Table S3, DOCX file, 0.02 MB. Copyright © 2021 Kang et al. 2021 Kang et al. https://creativecommons.org/licenses/by/4.0/ This content is distributed under the terms of the Creative Commons Attribution 4.0 International license.

### MGEs in the dental plaque among the HS group, PS group, and RS group. (i) The composition and correlation analysis of MGEs.

**FIG 6:**
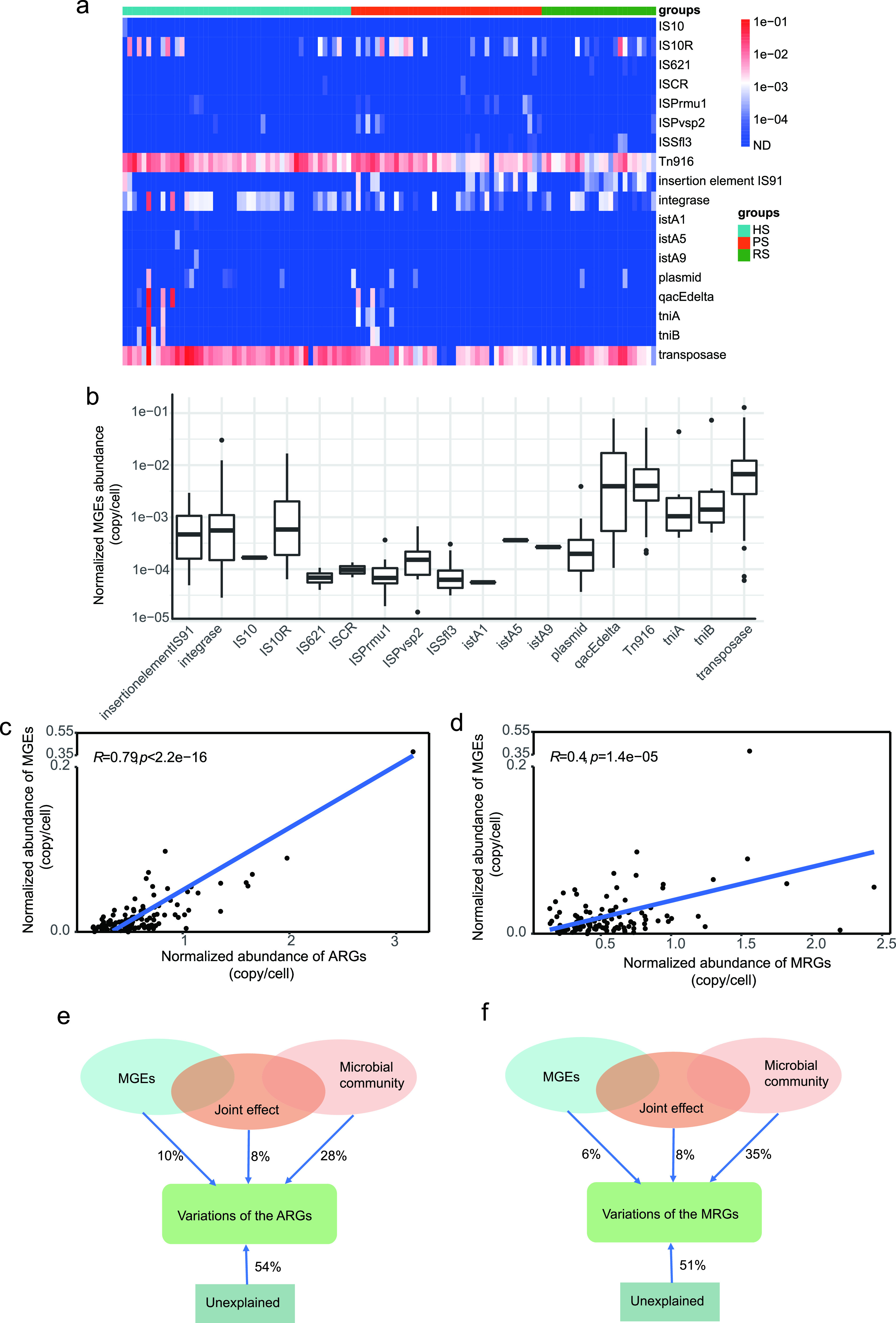
Composition and correlation analysis of MGEs. (a) Broad-spectrum quantitative profile of the MGE types (copies/cell) in 112 dental plaque samples. (b) Comparison of abundances of different MGE types in the 112 samples. (c and d) The total abundance of MGEs significantly correlated with the total abundance of detected ARGs (c) and the total abundance of detected MRGs (d) based on Pearson’s correlation. (e and d) pRDA differentiating the effect of microbial communities (at the phylum level) and MGEs on the profile of ARGs (e) and MRGs (f).

According to metagenomic analysis, we detected 18 MGE types in 111 of the 112 dental plaque samples. Transposase was composed of the greatest proportion of MGEs present in all samples, followed by Tn*916*. The abundance of the remaining classes of MGEs was relatively low (Fig. 6a and b). A total of 6 MGEs were significant positive correlations with the 10 of 44 putative ARG and/or MRG hosts. Specifically, Tn*916*-orf9 had a significant positive correlation with the relative abundance of *Bacteroidetes* oral taxon 274, Peptostreptococcus stomatis, Centipeda periodontii, Treponema denticola, Treponema maltophilum, Treponema *vincentii*, and Fretibacterium fastidiosum; Tn*916*-orf17 and Tn*916*-orf15 showed significant positive relationships with Fretibacterium fastidiosum; Tn*916*-orf16 showed significant positive relationships with *Bacteroidetes* oral taxon 274, Porphyromonas endodontalis, Peptostreptococcus stomatis, Dialister invisus, and Fretibacterium fastidiosum; IS91_CP001383.1 showed significant positive relationships with Lautropia mirabilis; abd transposase *tnpA* had significant positive relationships with *Bacteroidetes* oral taxon 274, Porphyromonas endodontalis, Treponema *vincentii*, and Fretibacterium fastidiosum (Fig. S6).

10.1128/mSphere.00162-21.6 FIG S6 Spearman correlations between 6 MGEs and 44 putative ARG and/or MRG hosts. The significant positive correlation is marked with an asterisk. (Benjamin-Hochberg method; *, *P* < 0.05; **, *P* < 0.01). Download FIG S6, PDF file, 0.4 MB. Copyright © 2021 Kang et al. 2021 Kang et al. https://creativecommons.org/licenses/by/4.0/ This content is distributed under the terms of the Creative Commons Attribution 4.0 International license.

The normalized abundance of MGEs was positively correlated with the normalized abundance of ARGs detected (Pearson's *r* = 0.79, *P* < 2.2e−16) (Fig. 6c) as well as the normalized abundance of MRGs (Pearson's *r* = 0.4, *P* < 1.4e−05) (Fig. 6d). We also evaluated the ability of MGEs and phylum-level microbial community composition to explain the relative abundance of ARGs and MRGs according to partial redundancy analysis (pRDA). The microbial community showed a greater contribution to the ARGs (Fig. 6e) and MRGs (Fig. 6f), explaining 28% and 35%, respectively. The MGEs accounted for 10% and 6% of the variation in the ARGs and MRGs, respectively. Microbial community attributes explained a higher fraction than the MGEs (ARGs, 28% versus 10%; MRGs, 35% versus 6%).

### (ii) Correlation network of cooccurring ARGs, MRGs, and MGEs.

**FIG 7:**
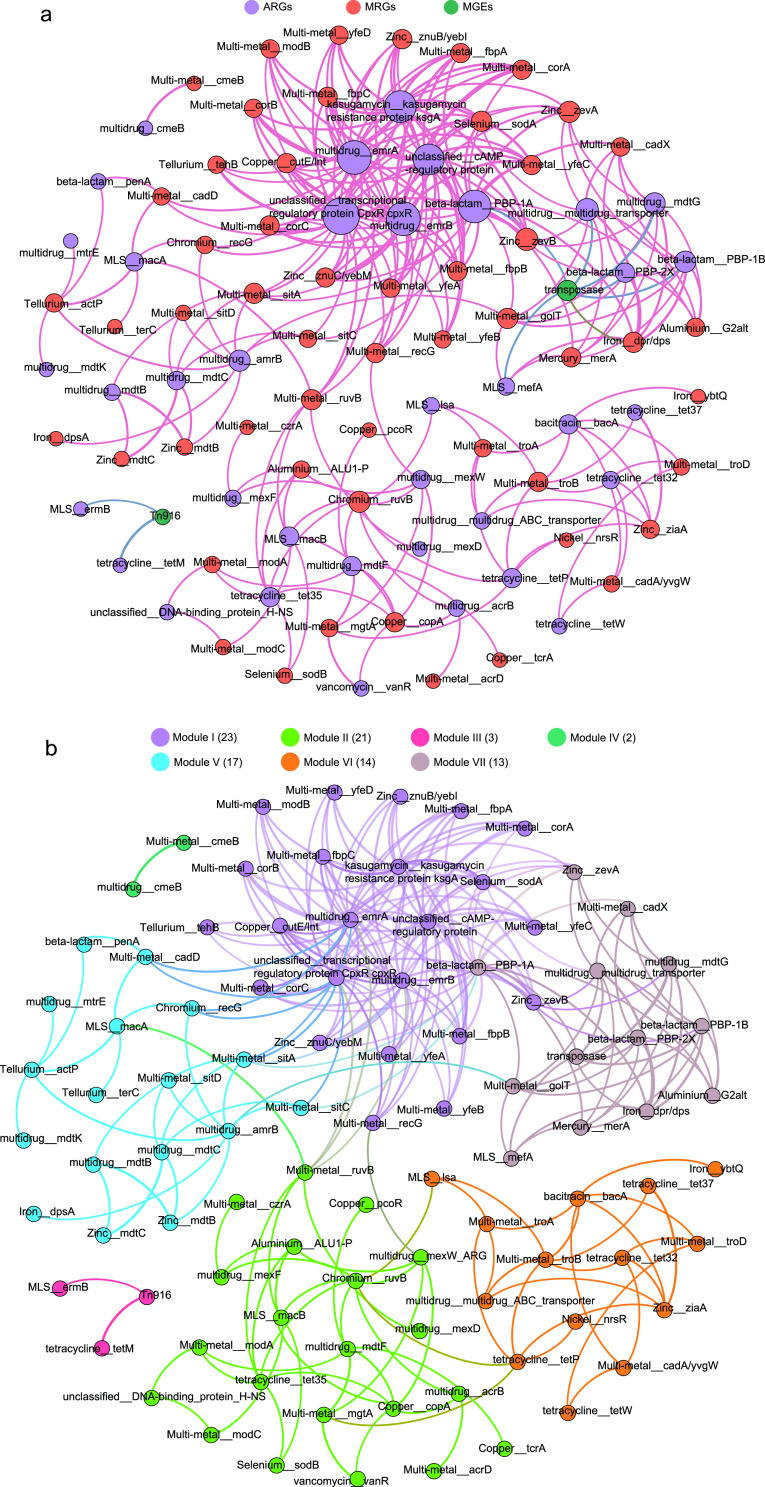
Network analysis showing the cooccurrence pattern between ARGs, MRGs, and MGEs based on Pearson’s correlation analysis. A connection represents a strong (Spearman’s *r*, >0.6) and significant (*P* < 0.01) correlation. The size of each node is proportional to the number of connections. (a) The nodes were colored according to ARGs, MRGs, and MGEs. (b) The nodes were colored according to modularity class.

To investigate the possibility of the cooccurrence of MRGs and ARGs on the same MGEs, we performed a network analysis of resistance genes. The resultant network consisted of 93 nodes (37 ARGs, 54 MRGs, and 2 MGEs) and 247 edges (Fig. 7a and b). We generated a total of seven modules based on the modularity class, and two of these modules were lightly connected. The modularity index of 0.571, calculated using Gephi’s modularity function (38), suggested that the network had a modular structure (39). We did not observe a cooccurrence of MRGs and ARGs in module III only. Of note, in module III, Tn*916* was significantly associated with *ermB* and *tetM*, which suggested that *ermB* and *tetM* could be transferred and cooccur by the conjugative transposon Tn*916*.

In modules I and VII, we found that ARGs and MRGs could be linked on the same MGEs. For example, the beta-lactam resistance genes (PBP-1A, PBP-1B, or PBP-2X) were specifically connected to iron resistance genes (*dpr-dps*) and transposase. The multidrug resistance genes (multidrug transporter ARG or *mdtG*) were significantly associated with iron resistance genes (*dpr-dps*) and transposase. The MLS resistance genes (*mefA*) occurred with iron resistance genes (*dpr-dps*) and transposase. As such, ARGs and MRGs possibly propagated along the same MGEs, increasing microbial resistance to antibiotics and metals.

## DISCUSSION

The rapid development and spread of antimicrobial resistance is a serious global public health problem that cannot be underestimated. Shotgun metagenomic sequencing technologies have been used as a screening tool for ARGs to monitor antibiotic resistance, such as in the human oral cavity (40), human (41) and animal (42, 43) gut, and in more extensive environments (44–46). Our study was the first to reveal the distribution of microbiome, ARGs, and MRGs in the dental plaque microbiota of subjects in HS, PS (before treatment), and RS (after treatment). To the best of our knowledge, despite several studies investigating the modification of the periodontal microflora in the PS, the distribution and cooccurrence of ARGs and MRGs have not been reported in the dental literature. Hence, monitoring the profile of these resistomes offers significant potential to provide instructions regarding proper use of antibiotics in patients with periodontitis and thereby to improve the actual efficacy of the treatment regimens.

We observed high microbial richness and diversity in the PS group compared with the HS group, which was consistent with the outcomes from past reports (47–49). Of concern, of the species enriched in the PS group (Fig. 1b), Tannerella forsythia, Treponema denticola, and Porphyromonas gingivalis, the so-called red complex pathogens cover the most important pathogens in adult periodontal disease. Campylobacter rectus, Streptococcus constellatus, Prevotella intermedia, and Fusobacterium nucleatum belong to the orange complex and serve as a bridge species between the early colonizers and highly pathogenic bacteria of the red complex (50). The colonization sites and the number of the red complex bacteria increase with an increase in colonization by the orange complex (51).

In an earlier study, Belstrøm et al. found that SRP treatment caused a significant decrease in the relative abundance of *Porphyromonas* and Treponema in combination with a significant increase in Streptococcus, *Rothia*, and *Actinomyces* in plaque samples (52). This was also identified similarly by subjects in our study. We found that the relative abundance of Porphyromonas endodontalis, Porphyromonas gingivalis, Treponema denticola, Treponema maltophilum, Treponema socranskii, Treponema *vincentii*, and Treponema medium decreased and the relative abundance of Streptococcus constellatus, Streptococcus sanguinis, Streptococcus mitis oralis pneumoniae, Rothia mucilaginosa, Actinomyces oris, Actinomyces viscosus, Rothia dentocariosa, and Actinomyces johnsonii increased significantly in the RS group. Of interest, a study on the effects of SRP treatment on salivary microorganisms has shown that despite the principally successful treatment outcome achieved during SRP treatment, the broad composition of the salivary microbiota is not influenced by the treatment during this period (53).

For nonbacterial components of the dental plaque microbiome, 10 of 14 virus species are phages, including Streptococcus, *Propionibacterium*, *Actinomyces*, and Pseudomonas phage, which indicated that bacteriophages are involved in shaping the microbial ecosystem of dental plaque (54). The fungus species Candida albicans was found in only 6 subjects. In one study, different types of *Candida*, especially Candida albicans, were isolated in the 35% of cultures of 55 subjects with periodontal disease. *Candida* may play an important role in adhering to soft tissues, allowing organisms to invade deeply (55). In our results, despite the low relative abundance of fungal species, the relative abundance of Candida albicans of 2 patients in the PS group was higher than that of 3 subjects in the HS group and 1 subject in the RS group.

Given that we did not observe any difference in the richness and diversity in the microbial community between the healthy and SRP groups, we expected the structure of the microbial communities to be similar in both groups, but this was not the case. A significant difference remained for the microbial community structure. This may have been because it takes awhile for microbial community structure to recover fully to a healthy state after SRP treatment. Additionally, the numbers of both ARGs and MRGs were greater in the PS group and SRP group than in the HS group.

During periodontitis progression, the changes in host physiology can affect dental plaque microbial community and their interaction pattern. In such a scenario, some species acting as key players in the community have been implicated as driver microbes (56). Among the driver microbes identified in this study, *T. forsythia* and *C. rectus* are the members of the red complex, and *F. fastidiosum* is associated with periodontitis (57). Of interest, *T. forsythia* was significantly more prevalent in periodontitis patients than healthy subjects. The detection frequency of *T. forsythia* also decreased significantly after SRP, which was consistent with previous findings by Wadhwani et al. (58). Tannerella forsythia is an important pathogen associated with periodontitis that can initiate gingival tissue destruction and alveolar bone resorption in periodontal disease (59). The current body of evidence suggests the strong relationship of Tannerella forsythia with the most meaningful clinical parameters of periodontal diagnosis, that is, pocket probing depth and bleeding (50, 60). Our results demonstrated that *T. forsythia* is a major driver microbe of periodontitis progression, and it can be reduced by SRP treatment to nearly normal levels.

As potent antimicrobial agents, dental surgeons frequently prescribe antibiotics for therapeutic and prophylactic reasons (61). Commonly used antibiotics in clinical dentistry include amoxicillin (beta-lactam), amoxicillin-clavulanic acid (beta-lactam), azithromycin (macrolide), clindamycin (lincomycin), ciprofloxacin (quinolone), gentamicin (aminoglycoside), metronidazole (nitroimidazole), penicillin (beta-lactam), and tetracycline (tetracycline) (11, 62). Note that bacitracin, beta-lactam, MLS, multidrug, and tetracycline resistance genes existed in almost all dental plaque samples at relatively high abundances. Carr et al. showed that MLS, tetracycline, cephamycin, fluoroquinolone, glycylcycline, and pleuromutilin resistance genes were present in the dental plaque of more than half of the healthy subjects (40). This may partly explain the declining efficacy of clinically used antibiotics and more frequent antibiotic treatment failures. Multidrug-resistant bacteria are a leading public health issue. In the context of the rapid growth of antimicrobial resistance, the emergence and infections by multidrug-resistant bacteria have increased at an alarming rate. The increase in multidrug-resistant genes in the oral microbiome has emphasized the necessity for the appropriate and correct use of antibiotics in dental practice (4, 63, 64).

The results of Procrustes analysis and pRDA suggested that the microbial community is a key determinant of antibiotic and metal resistance. Our results were broadly consistent with the phenomenon presented by Forsberg et al., i.e., the bacterial phylogeny may shape the resistome when a strong correlation is observed between the ARG composition and bacterial community, whereas the horizontal gene transfer (HGT) is kept at a low frequency (65). Analyzing the cooccurrence patterns of microbial taxa and resistance genes, we speculated that 32 species were possible hosts of 25 ARG subtypes and speculated that 35 species were possible hosts of 40 ARG subtypes. These ARG- and MRG-carrying pathogens, with the ability to withstand antibiotics, pose a high risk to human health (41). Therefore, special attention should be given to these bacteria to control or reduce the risk of treatment failure.

Recent studies based on culture and metagenomics have revealed that tetracycline-resistant gene *tet*(M) predominated among the detected tet genes (66–68), and our study also found a high abundance of *tet*(M). Our results also showed that the *tet*(M) gene was significantly linked with *ermB* (erythromycin-resistant gene) and Tn*916*. We often observed the *tet*(M) gene and *erm* gene to be contained on the Tn*916* conjugative transposon (69, 70). Therefore, the usage of erythromycin may coselect for tetracycline-resistant bacteria (71). *tet*(M) has been shown to display 95% nucleotide sequence similarity in a series of bacteria, hinting that this gene was acquired through HGT (72, 73). By PCR and southern blot analysis, Lancaster et al. found that the *tet*(M) gene is located on a Tn*916*-like element in the oral cavity of children (74). The prevalence of tetracycline-resistant genes in the dental plaque resistome may be explained by coselection.

Previous studies have reported that the same ARGs or MRGs could be cotransferred through the same or different conjugative MGEs (75, 76). Transposase, one of the important MGEs, was significantly linked to ARGs and MRGs in our network analysis. It has been suggested that the nature of the oral biofilm is a conducive environment for complex bacterial interactions, including HGT (72, 73). Increased content of heavy metals in the environment has been demonstrated to coregulate genes responsible for antibiotic resistance and have reduced sensitivity to antibiotics (26, 77). Taken together, the coselection phenomenon can contribute to the dissemination and maintenance of antibiotic resistance, thereby posing a major public health problem. This emphasizes the necessity for clinically reasonable and effective use of metallic nanomaterials for antimicrobials. However, the cooccurrence network was based on statistical analyses. Therefore, further studies using other types of methodologies are required to obtain more accurate predictions of the cooccurrence of ARGs, MRGs, and MGEs.

The results of this study indicated significant alteration of the profiles of the microbial community, ARGs, and MRGs in the dental plaque because of periodontitis and SRP treatment. The changes in resistomes were significantly related to the microbial community, which suggested that microbial community plays a key role in controlling the dispersal of ARGs and MRGs. The cooccurrence of ARGs, MRGs, and MGEs may help the microbes to increase their resistance, but further experiments are required to verify this interpretation.

## MATERIALS AND METHODS

### Data collection and information.

To better understand the composition of the resistance genes in dental plaque samples in HS, PS (before treatment), and RS (after treatment) obtained from the NCBI SRA database (https://www.ncbi.nlm.nih.gov/sra) under NCBI BioProject no. PRJNA255922 (no publication), PRJNA528558 (2), PRJNA625082 (78), and PRJNA230363 (79), we downloaded a total of 112 dental plaque metagenome data sets with a read length of ≥100. These included data sets from 48 healthy individuals, 40 individuals with periodontitis, and 24 individuals with the RS. The detailed accession number is provided in Table S1 in the supplemental material. The basic information of subjects is described in Text S1. We transformed the FASTQ file from the SRA format using the command fastp-dump, which is included in the NCBI SRA Toolkit (www.ncbi.nlm.nih.gov/sra).

10.1128/mSphere.00162-21.7 TEXT S1 Subject information. Download Text S1, DOCX file, 0.01 MB. Copyright © 2021 Kang et al. 2021 Kang et al. https://creativecommons.org/licenses/by/4.0/ This content is distributed under the terms of the Creative Commons Attribution 4.0 International license.

10.1128/mSphere.00162-21.8 TABLE S1 Basic information of the 112 collected datasets. Download Table S1, DOCX file, 0.02 MB. Copyright © 2021 Kang et al. 2021 Kang et al. https://creativecommons.org/licenses/by/4.0/ This content is distributed under the terms of the Creative Commons Attribution 4.0 International license.

### Bioinformatics analysis. (i) Microbial community and pathogens analysis.

The human sequences (according to alignment to hg19) were discarded along with low-quality reads. The high-quality sequences were taxonomically classified using MetaPhlAn2 software with default parameters, with a database comprising ∼17,000 reference genomes (∼13,500 bacterial and archaeal, ∼3,500 viral, and ∼110 eukaryotic) (80). We performed principal coordinate analysis (PCoA) of Bray-Curtis and Jaccard distances among profiles at the species level using the vegan package in the R environment. Inverse Simpson and Shannon diversity indices of the samples were calculated at the species level using the vegan function diversity. We based identification of driver species between the case and control on NetShift workflow (56).

### (ii) ARGs, MGEs, and BMRGs analysis.

We identified and annotated ARG-like reads in all samples using the protocol followed by Yin et al. (33). The default parameters for ARGs annotation were an E value of ≤1 × 10^−7^, ≥80% sequence identity, and ≥25 amino acids of alignment length. The core database of ARGs-OAP was the Structured Antibiotic Resistance Genes (SARG) database, which contained 24 ARG types (e.g., tetracycline-resistant gene) and 1,244 ARG subtypes (e.g., *tetA* and *tetB*). The SARG was mainly derived from a combination of the Antibiotic Resistance Database (ARDB; http://ardb.cbcb.umd.edu/) and the Comprehensive Antibiotic Resistance Database (CARD; https://card.mcmaster.ca/). We used customized Perl scripts to prescreen ARG-like and 16S rRNA gene sequences. We calculated and recorded ARG abundance (copies of ARG per cell) using the following equation:

$$\text{Abundance}=\sum_{i}^{n}\frac{N_{i\left(\text{ARG-like sequence}\right)}\times L_{\text{reads}}/L_{i\left(\text{ARGs reference sequence}\right)}}{N_{16\text{S sequence}}\times L_{\text{reads}}/L_{\text{16S sequence}}}\times N_{\text{16S copy number}}$$

where *N*_i(ARG-like sequence)_ is the number of the ARG-like reads annotated with one specific ARG reference sequence; *L_i_*_(ARGs reference sequence)_ is the sequence length of the corresponding specific ARG reference sequence; *N*_16S sequence_ is the number of the 16S rRNA gene sequence identified from the metagenomic data by sequence similarity alignment to the Greengenes database (32); *L*_16S sequence_ is the mean sequence length of 16S rRNA genes (1,432 bp) in the Greengenes database (81); *n* is the number of the mapped ARG reference sequence belonging to the ARG type or subtype; *L*_reads_ is the sequence length of the Illumina reads that was used in our study; and *N*_16S copy number_ is the mean copy number of 16S rRNA genes per cell. The mean copy number within cells was calculated by using CopyRighter (82).

We searched the metagenomic sequences for MRGs against the experimentally confirmed MRGs in the BacMet database (version 2.0) (http://bacmet.biomedicine.gu.se) using BLASTX with an E value of ≤1 × 10^−7^ (83). A read was annotated as an MRG-like fragment if the sequence identity was ≥80% and the alignment length was ≥25 amino acids. We calculated and recorded MRG abundance (copies of MRG per cell) using the following equation:

$$\text{Abundance}=\sum_{i}^{n}\frac{N_{i\left(\text{MRG-like sequence}\right)}\times L_{\text{reads}}/L_{i\left(\text{MRGs reference sequence}\right)}}{N_{\text{16S sequence}}\times L_{\text{reads}}/L_{16\text{S sequence}}}\times N_{\text{16S copy number}}$$

where *N_i_*_(MRG-like sequence)_ is the number of the MRG-like reads annotated with one specific MRG reference sequence; *L_i_*_(MRGs reference sequence)_ is the sequence length of the corresponding specific MRG reference sequence; *N*_16S sequence_ is the number of the 16S rRNA gene sequence identified from the metagenomic data by sequence similarity alignment to Greengenes database (32); *L*_16S sequence_ is the mean sequence length of 16S rRNA genes (1,432 bp) in the Greengenes database (81); *n* is the number of the mapped MRG reference sequence belonging to the MRG type or subtype; *L*_reads_ is the sequence length of the Illumina reads that was used in our study; and *N*_16S copy number_ is the mean copy number of 16S rRNA genes per cell. The mean copy number within cells was calculated by using CopyRighter (82).

We searched all of the clean data for MGEs against a custom MGE database (https://github.com/KatariinaParnanen/MobileGeneticElementDatabase) (84), which was created by fetching coding sequences for genes that were annotated as IS*, ISCR*, intI1, int2, istA*, istB*, qacEdelta, tniA*, tniB*, tnpA*, or Tn*916* transposon open reading frames, or genes in the NCBI nucleotide database (85), and also included the PlasmidFinder database (86). If a sequence identity was ≥80% over an alignment length of ≥25 amino acids with an E value of ≤1 × 10^−7^, we annotated it as MGE. We calculated and recorded MGE abundance (copies of MGE per cell) using the following equation:

$$\text{Abundance}=\sum_{i}^{n}\frac{N_{i\left(\text{MGE-like sequence}\right)}\times L_{\text{reads}}/L_{i\left(\text{MGEs reference sequence}\right)}}{N_{\text{16S sequence}}\times L_{\text{reads}}/L_{\text{16S sequence}}}\times N_{\text{16S copy number}}$$

where *N_i_*_(MRG-like sequence)_ is the number of the MGE-like reads annotated with one specific MGE reference sequence; *L_i_*_(MRGs reference sequence)_ is the sequence length of the corresponding specific MGE reference sequence; *N*_16S sequence_ is the number of the 16S rRNA gene sequence identified from the metagenomic data by sequence similarity alignment to the Greengenes database (32); *L*_16S sequence_ is the mean sequence length of 16S rRNA genes (1,432 bp) in the Greengenes database (81); *n* is the number of the mapped MGE reference sequence belonging to the MGE type or subtype; *L*_reads_ is the sequence length of the Illumina reads that was used in our study; *N*_16S copy number_ is the mean copy number of 16S rRNA genes per cell. The mean copy number within cells was calculated by using CopyRighter (82).

### Statistical analysis.

We conducted alpha and beta diversity indices, PCoA, procrustes analysis, pRDA, and permutational multivariate analysis of variance (PERMANOVA) using the vegan package in R. We used the Kruskal-Wallis test to compare three groups and applied the Wilcoxon rank-sum test to verify statistical significance between two groups. We considered a *P* value corrected by the Benjamini-Hochberg procedure of less than 0.05 as a statistically significant difference. We performed Pearson’s correlations with the R package ggpubr. We calculated all Spearman’s rank correlation coefficients of network analyses in R with Hmisc packages. We realized the network visualization using the Gephi platform (https://gephi.org/) and created figures in R (version 4.0.2).

### Availability of data and materials.

The metagenomic data are available under NCBI BioProject numbers PRJNA255922, PRJNA528558, PRJNA625082, and PRJNA230363.
