# Supplementary figures and images for: Dental Plaque Microbial Resistomes of Periodontal Health and Disease and Their Changes after Scaling and Root Planing Therapy
Source: mSphere. 2021 Jul 21;6(4):e00162-21. doi: 10.1128/mSphere.00162-21 (PMC8386447; doi:10.1128/mSphere.00162-21)

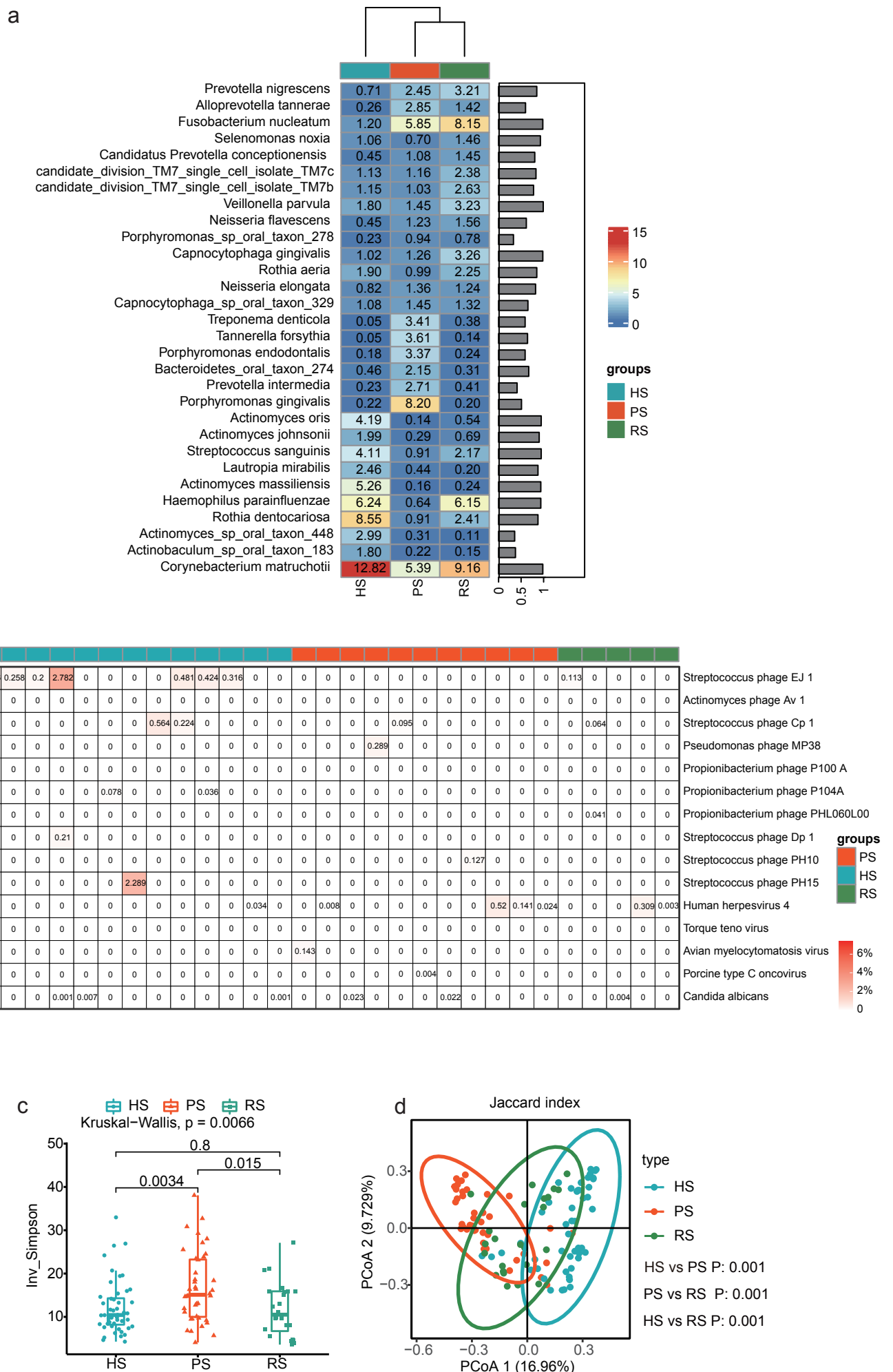

Figure.S1

Supplement: FIG S1 [file msphere.00162-21-sf001.pdf]

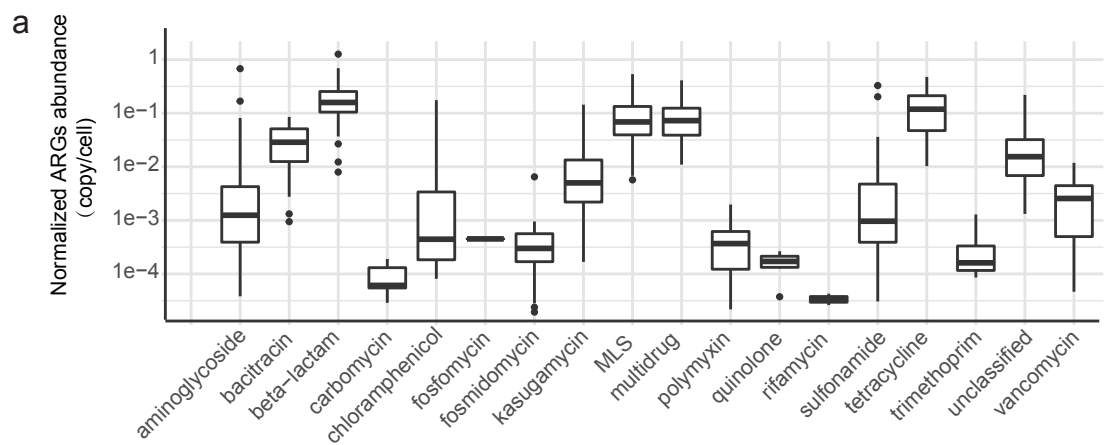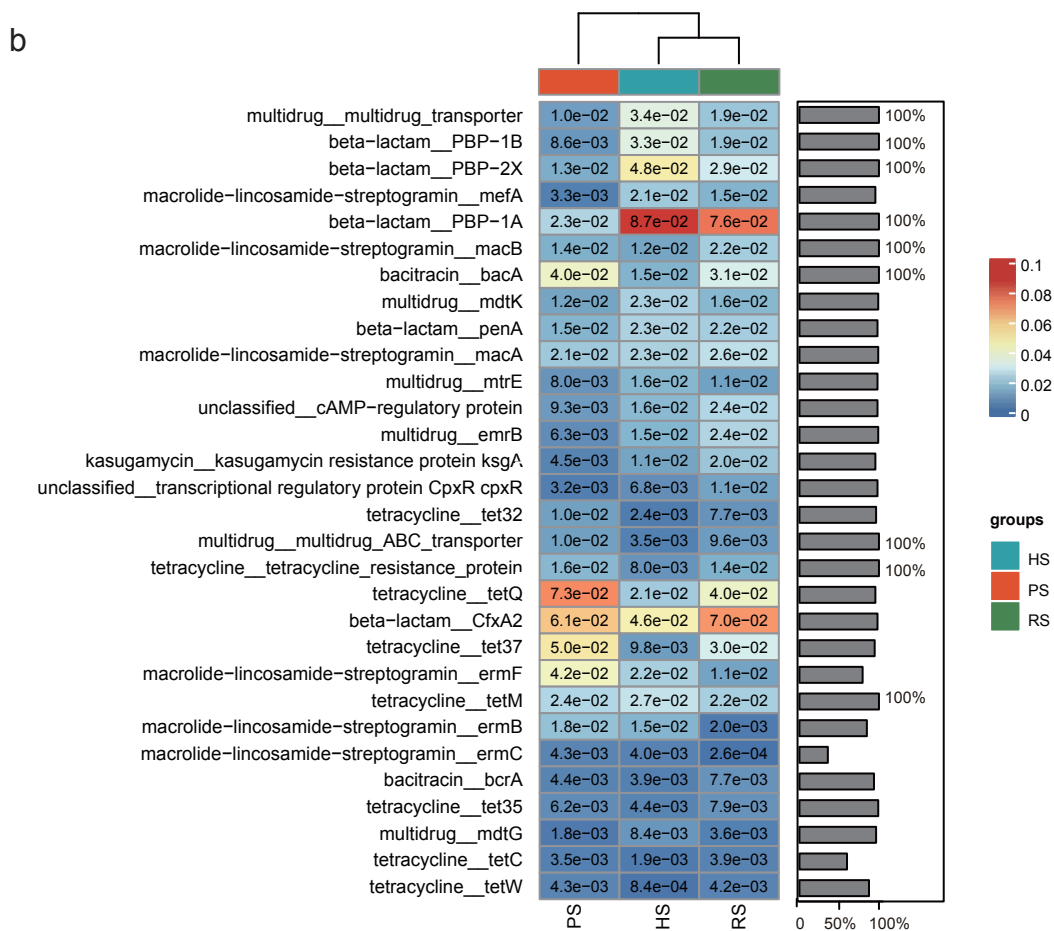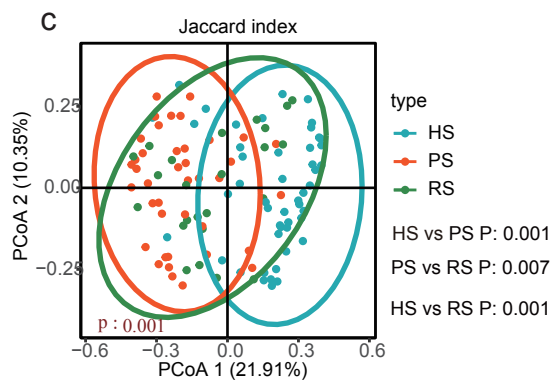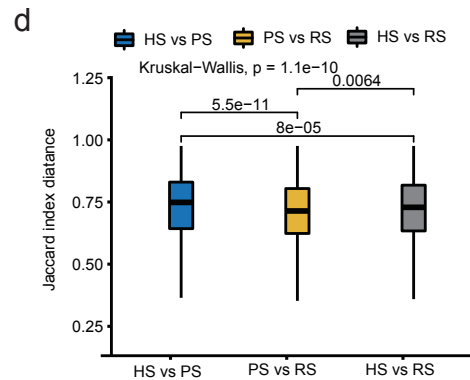

Figure.S2

Supplement: FIG S2 [file msphere.00162-21-sf002.pdf]

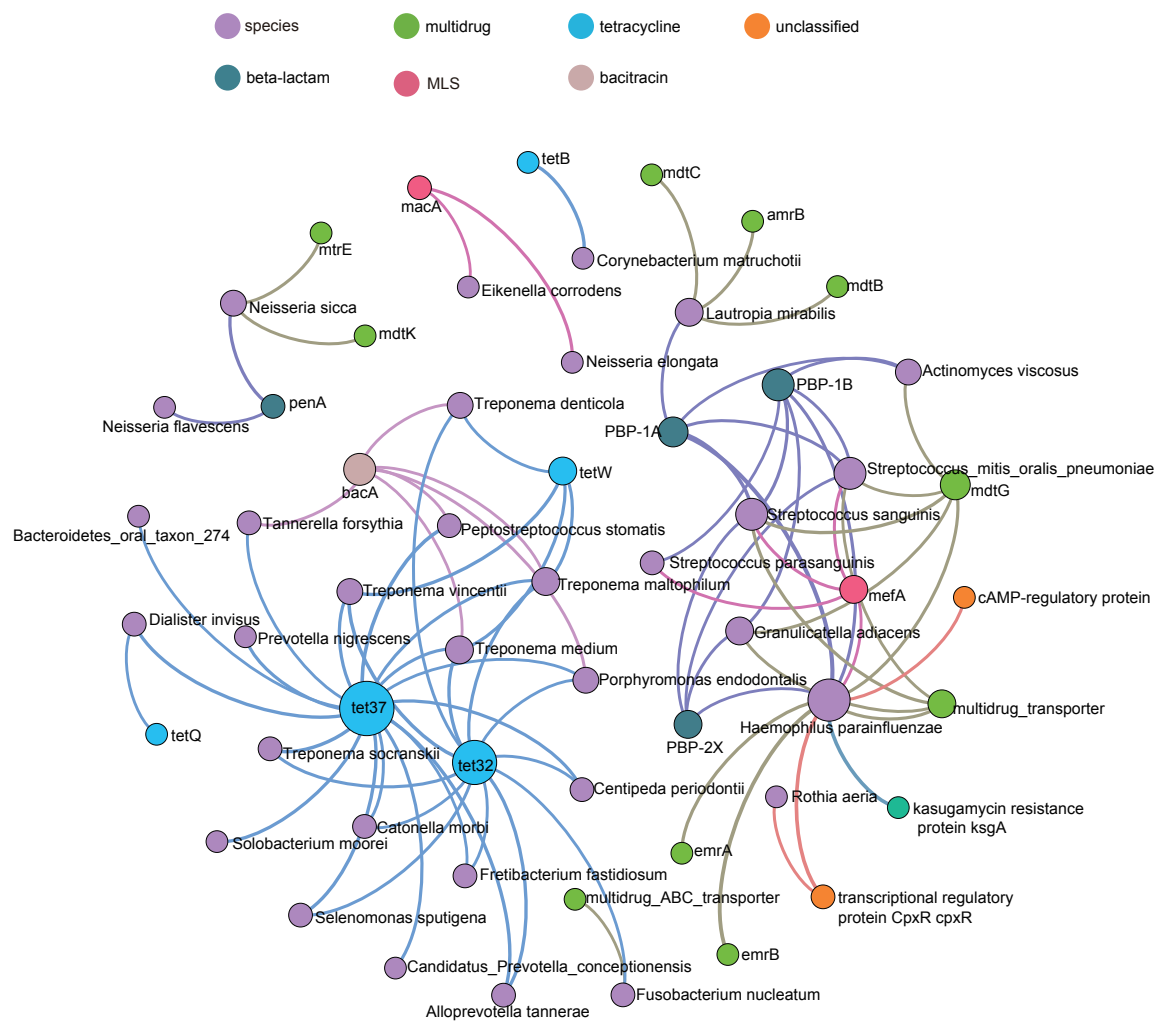

Figure.S3

Supplement: FIG S3 [file msphere.00162-21-sf003.pdf]

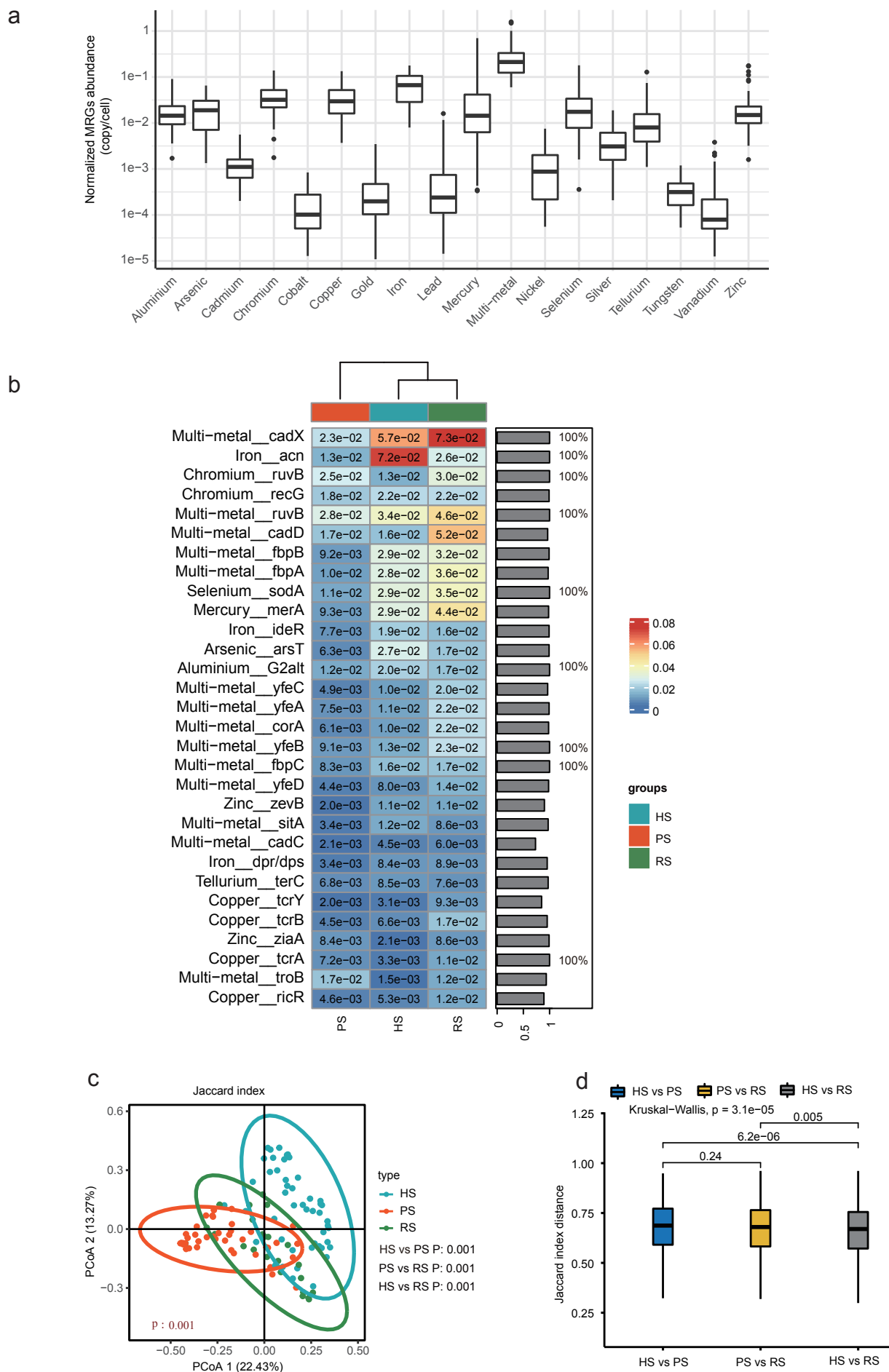

Figure.S4

Supplement: FIG S4 [file msphere.00162-21-sf004.pdf]

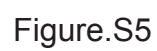

Figure.S5

Supplement: FIG S5 [file msphere.00162-21-sf005.pdf]

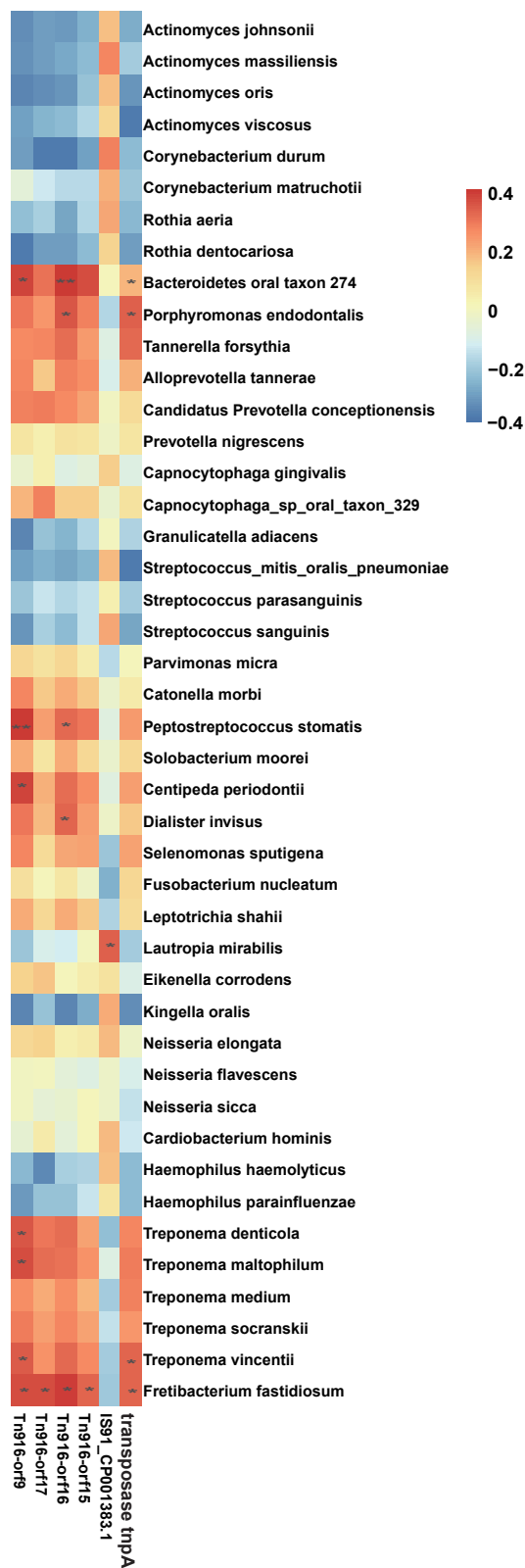

Figure.S6

Supplement: FIG S6 [file msphere.00162-21-sf006.pdf]
