# Supplementary material for: Dental Plaque Microbial Resistomes of Periodontal Health and Disease and Their Changes after Scaling and Root Planing Therapy
Source: mSphere. 2021 Jul 21;6(4):e00162-21. doi: 10.1128/mSphere.00162-21 (PMC8386447; doi:10.1128/mSphere.00162-21)
Supplement: TABLE S2 [file msphere.00162-21-st002.docx]

**Table S2** Potential ARG hosts information revealed by co-occurrence between ARG subtypes and microbial taxa.

| Species | Phylum | ARG subtype | ARG type |
| --- | --- | --- | --- |
| Haemophilus parainfluenzae | Proteobacteria | PBP-1A | beta-lactam |
|  |  | PBP-1B | beta-lactam |
|  |  | PBP-2X | beta-lactam |
|  |  | kasugamycin resistance protein ksgA | kasugamycin |
|  |  | mefA | MLS |
|  |  | emrA | multidrug |
|  |  | emrB | multidrug |
|  |  | mdtG | multidrug |
|  |  | Multidrug transporter | multidrug |
|  |  | cAMP-regulatory protein | unclassified |
|  |  | transcriptional regulatory protein CpxR cpxR | unclassified |
| Streptococcus sanguinis | Firmicutes | PBP-1A | beta-lactam |
|  |  | PBP-1B | beta-lactam |
|  |  | PBP-2X | beta-lactam |
|  |  | mefA | MLS |
|  |  | mdtG | multidrug |
|  |  | Multidrug transporter | multidrug |
| Streptococcus_mitis_oralis_pneumoniae | Firmicutes | PBP-1A | beta-lactam |
|  |  | PBP-1B | beta-lactam |
|  |  | PBP-2X | beta-lactam |
|  |  | mefA | MLS |
|  |  | mdtG | multidrug |
|  |  | Multidrug transporter | multidrug |
| Treponema medium | Spirochaetes | bacA | bacitracin |
|  |  | tet32 | tetracycline |
|  |  | tet37 | tetracycline |
|  |  | tetW | tetracycline |
| Treponema maltophilum | Spirochaetes | bacA | bacitracin |
|  |  | tet32 | tetracycline |
|  |  | tet37 | tetracycline |
|  |  | tetW | tetracycline |
| Lautropia mirabilis | Proteobacteria | PBP-1A | beta-lactam |
|  |  | amrB | multidrug |
|  |  | mdtB | multidrug |
|  |  | mdtC | multidrug |
| Granulicatella adiacen | Firmicutes | PBP-1B | beta-lactam |
|  |  | PBP-2X | beta-lactam |
|  |  | mdtG | multidrug |
|  |  | Multidrug transporter | multidrug |
| Treponema vincentii | Spirochaetes | tet32 | tetracycline |
|  |  | tet37 | tetracycline |
|  |  | tetW | tetracycline |
| Treponema denticola | Spirochaetes | bacA | bacitracin |
|  |  | tet32 | tetracycline |
|  |  | tetW | tetracycline |
| Porphyromonas endodontalis | Bacteroidetes | bacA | bacitracin |
|  |  | tet32 | tetracycline |
|  |  | tet37 | tetracycline |
| Neisseria sicca | Proteobacteria | penA | beta-lactam |
|  |  | mdtK | multidrug |
|  |  | mtrE | multidrug |
| Actinomyces viscosus | Actinobacteria | PBP-1A | beta-lactam |
|  |  | PBP-1B | beta-lactam |
|  |  | mdtG | multidrug |
| Treponema socranskii | Spirochaetes | tet32 | tetracycline |
|  |  | tet37 | tetracycline |
| Tannerella forsythia | Bacteroidetes | bacA | bacitracin |
|  |  | tet37 | tetracycline |
| Streptococcus parasanguinis | Firmicutes | PBP-1B | beta-lactam |
|  |  | mefA | MLS |
| Selenomonas sputigena | Firmicutes | tet32 | tetracycline |
|  |  | tet37 | tetracycline |
| Peptostreptococcus stomatis | Firmicutes | bacA | bacitracin |
|  |  | tet37 | tetracycline |
| Fusobacterium nucleatum | Fusobacteria | Multidrug ABC transporter | multidrug |
|  |  | tet32 | tetracycline |
| Fretibacterium fastidiosum | Synergistetes | tet32 | tetracycline |
|  |  | tet37 | tetracycline |
| Dialister invisus | Firmicutes | tet37 | tetracycline |
|  |  | tetQ | tetracycline |
| Centipeda periodontii | Firmicutes | tet32 | tetracycline |
|  |  | tet37 | tetracycline |
| Catonella morbi | Firmicutes | tet32 | tetracycline |
|  |  | tet37 | tetracycline |
| Alloprevotella tannerae | Bacteroidetes | tet32 | tetracycline |
|  |  | tet37 | tetracycline |
| Solobacterium moorei | Firmicutes | tet38 | tetracycline |
| Rothia aeria | Actinobacteria | transcriptional regulatory protein CpxR cpxR | unclassified |
| Prevotella nigrescens | Bacteroidetes | tet37 | tetracycline |
| Neisseria flavescens | Proteobacteria | penA | beta-lactam |
| Neisseria elongata | Proteobacteria | macA | MLS |
| Eikenella corrodens | Proteobacteria | macA | MLS |
| Corynebacterium matruchotii | Actinobacteria | tetB | tetracycline |
| Candidatus Prevotella conceptionensis | Bacteroidetes | tet37 | tetracycline |
| Bacteroidetes_oral_taxon_274 | Bacteroidetes | tet37 | tetracycline |
