# Supplementary material for: Dental Plaque Microbial Resistomes of Periodontal Health and Disease and Their Changes after Scaling and Root Planing Therapy
Source: mSphere. 2021 Jul 21;6(4):e00162-21. doi: 10.1128/mSphere.00162-21 (PMC8386447; doi:10.1128/mSphere.00162-21)
Supplement: TABLE S3 [file msphere.00162-21-st003.docx]

**Table S3** Potential MRG hosts information revealed by co-occurrence between MRG subtypes and microbial taxa.

| Species | Phylum | ARG subtype | ARG type |
| --- | --- | --- | --- |
| Haemophilus parainfluenzae | Proteobacteria | cutE/lnt | Copper |
|  |  | merA | Mercury |
|  |  | corA | Multi-metal |
|  |  | corC | Multi-metal |
|  |  | fbpA | Multi-metal |
|  |  | fbpB | Multi-metal |
|  |  | fbpC | Multi-metal |
|  |  | golT | Multi-metal |
|  |  | sitA | Multi-metal |
|  |  | sitC | Multi-metal |
|  |  | sitD | Multi-metal |
|  |  | yfeC | Multi-metal |
|  |  | yfeD | Multi-metal |
|  |  | sodA | Selenium |
|  |  | zevA | Zinc |
|  |  | zevB | Zinc |
|  |  | znuB/yebI | Zinc |
|  |  | znuC/yebM | Zinc |
| Lautropia mirabilis | Proteobacteria | merT | Mercury |
|  |  | golT | Multi-metal |
|  |  | sitA | Multi-metal |
|  |  | sitC | Multi-metal |
|  |  | sitD | Multi-metal |
|  |  | mdtB | Zinc |
|  |  | mdtC | Zinc |
|  |  | zevB | Zinc |
| Streptococcus sanguinis | Firmicutes | merA | Mercury |
|  |  | merT | Mercury |
|  |  | cadX | Multi-metal |
|  |  | golT | Multi-metal |
|  |  | sitC | Multi-metal |
|  |  | zevA | Zinc |
|  |  | zevB | Zinc |
| Treponema socranskii | Spirochaetes | ybtQ | Iron |
|  |  | cadA/yvgW | Multi-metal |
|  |  | troB | Multi-metal |
|  |  | troD | Multi-metal |
|  |  | ziaA | Zinc |
| Treponema medium | Spirochaetes | ybtQ | Iron |
|  |  | cadA/yvgW | Multi-metal |
|  |  | troB | Multi-metal |
|  |  | troD | Multi-metal |
|  |  | ziaA | Zinc |
| Treponema maltophilum | Spirochaetes | ybtQ | Iron |
|  |  | cadA/yvgW | Multi-metal |
|  |  | troB | Multi-metal |
|  |  | troD | Multi-metal |
|  |  | ziaA | Zinc |
| Rothia dentocariosa | Actinobacteria | arsT | Arsenic |
|  |  | acn | Iron |
|  |  | ideR | Iron |
|  |  | merA | Mercury |
|  |  | zevA | Zinc |
| Actinomyces oris | Actinobacteria | arsT | Arsenic |
|  |  | acn | Iron |
|  |  | ideR | Iron |
|  |  | golT | Multi-metal |
|  |  | zevA | Zinc |
| Neisseria elongata | Proteobacteria | copF | Copper |
|  |  | nczA | Multi-metal |
|  |  | actP | Tellurium |
|  |  | terC | Tellurium |
| Fretibacterium fastidiosum | Synergistetes | ybtQ | Iron |
|  |  | cadA/yvgW | Multi-metal |
|  |  | troB | Multi-metal |
|  |  | troD | Multi-metal |
| Eikenella corrodens | Proteobacteria | copF | Copper |
|  |  | dpsA | Iron |
|  |  | cnrA | Multi-metal |
|  |  | terC | Tellurium |
| Corynebacterium durum | Actinobacteria | arsT | Arsenic |
|  |  | acn | Iron |
|  |  | ideR | Iron |
|  |  | sitD | Multi-metal |
| Actinomyces massiliensis | Actinobacteria | arsT | Arsenic |
|  |  | acn | Iron |
|  |  | ideR | Iron |
|  |  | silP | Silver |
| Treponema vincentii | Spirochaetes | cadA/yvgW | Multi-metal |
|  |  | troD | Multi-metal |
|  |  | ziaA | Zinc |
| Treponema denticola | Spirochaetes | cadA/yvgW | Multi-metal |
|  |  | troB | Multi-metal |
|  |  | troD | Multi-metal |
| Tannerella forsythia | Bacteroidetes | cadA/yvgW | Multi-metal |
|  |  | troB | Multi-metal |
|  |  | troD | Multi-metal |
| Rothia aeria | Actinobacteria | sitA | Multi-metal |
|  |  | sitC | Multi-metal |
|  |  | sitD | Multi-metal |
| Fusobacterium nucleatum | Fusobacteria | troB | Multi-metal |
|  |  | troD | Multi-metal |
|  |  | ziaA | Zinc |
| Corynebacterium matruchotii | Actinobacteria | ctpG | Copper |
|  |  | acn | Iron |
|  |  | ideR | Iron |
| Porphyromonas endodontalis | Bacteroidetes | cadA/yvgW | Multi-metal |
|  |  | troD | Multi-metal |
| Peptostreptococcus stomatis | Firmicutes | troD | Multi-metal |
|  |  | ziaA | Zinc |
| Granulicatella adiacens | Firmicutes | cadC | Multi-metal |
|  |  | cadX | Multi-metal |
| Cardiobacterium hominis | Proteobacteria | dpsA | Iron |
|  |  | terC | Tellurium |
| Capnocytophaga gingivalis | Bacteroidetes | copF | Copper |
|  |  | terC | Tellurium |
| Alloprevotella tannerae | Bacteroidetes | troD | Multi-metal |
|  |  | ziaA | Zinc |
| Actinomyces viscosus | Actinobacteria | arsT | Arsenic |
|  |  | acn | Iron |
| Actinomyces johnsonii | Actinobacteria | arsT | Arsenic |
|  |  | acn | Iron |
| Streptococcus_mitis_oralis_pneumoniae | Firmicutes | zevA | Zinc |
| Selenomonas sputigena | Firmicutes | ziaA | Zinc |
| Parvimonas micra | Firmicutes | ziaA | Zinc |
| Leptotrichia shahii | Fusobacteria | copB | Copper |
| Kingella oralis | Proteobacteria | acn | Iron |
| Haemophilus haemolyticus | Proteobacteria | zevA | Zinc |
| Capnocytophaga_sp_oral_taxon_329 | Bacteroidetes | nczA | Multi-metal |
| Bacteroidetes_oral_taxon_274 | Bacteroidetes | troD | Multi-metal |
