# Supplementary material for: Dental Plaque Microbial Resistomes of Periodontal Health and Disease and Their Changes after Scaling and Root Planing Therapy
Source: mSphere. 2021 Jul 21;6(4):e00162-21. doi: 10.1128/mSphere.00162-21 (PMC8386447; doi:10.1128/mSphere.00162-21)
Supplement: TEXT S1 [file msphere.00162-21-s0001.docx]

**Text S1**

In Bioproject PRJNA255922, before and after SRP treatment, 48 dental plaque samples were sampled from 12 periodontitis subjects whose two affected tooth sites per subject on average were sampled. The 12 subjects were all non-smokers, whose ages ranged from 37 to 65 years with an average age of 53 years. The authors of corresponding published article and uploaders of metagenomic data did not provide the information of subjects’ gender. These patients had a second clinic visit 4 to 19 weeks (on average 60 days) after completion of initial therapy. The sites of nine subjects had resolved at the second clinic visit, and the same tooth sites were resampled. Three of the 12 subjects were followed up with a third clinic visit 7 to 14 months after the second visit, and the same tooth sites had resolved and resampled.

In Bioproject PRJNA528558, the corresponding article hasn’t been published, and uploaders of metagenomic data did not provide the information of subjects’ gender and age, and smoking status.

In Bioproject PRJNA625082, 35 dental plaque samples were sampled from seven healthy subjects, 4 female, 3 male, in the age range 21 to 55 years. Subjects were sampled 5 to 6 times over a 5- to 9-day period. The authors of corresponding published article and uploaders of metagenomic data did not provide the information of subjects’ smoking status.

In Bioproject PRJNA230363, 20 dental plaque samples were sampled from 10 periodontitis patients and 10 periodontally healthy subjects. The 20 subjects were all non-smokers. The authors of corresponding published article and uploaders of metagenomic data did not provide the information of subjects’ age and gender.
