# Supplementary material for: Dental Plaque Microbial Resistomes of Periodontal Health and Disease and Their Changes after Scaling and Root Planing Therapy
Source: mSphere. 2021 Jul 21;6(4):e00162-21. doi: 10.1128/mSphere.00162-21 (PMC8386447; doi:10.1128/mSphere.00162-21)
Supplement: TABLE S1 [file msphere.00162-21-st001.docx]

**Table S1** Basic information of the 112 collected datasets.

| BioProject | Study condition | | NCBI accession | Data type | Region | Reads length (bp) | Base pairs （bp） |
| --- | --- | --- | --- | --- | --- | --- | --- |
| PRJNA255922 | | periodontitis | SRR1781914 | Illumina | United States | 100 | 5794329769 |
| PRJNA255922 | | periodontitis | SRR1781969 | Illumina | United States | 100 | 4041845567 |
| PRJNA255922 | | SRP | SRR1781970 | Illumina | United States | 100 | 939139011 |
| PRJNA255922 | | SRP | SRR1781983 | Illumina | United States | 100 | 5567332395 |
| PRJNA255922 | | periodontitis | SRR1779468 | Illumina | United States | 100 | 1273692559 |
| PRJNA255922 | | SRP | SRR1779479 | Illumina | United States | 100 | 1761322406 |
| PRJNA255922 | | periodontitis | SRR1779482 | Illumina | United States | 100 | 1378842336 |
| PRJNA255922 | | periodontitis | SRR1779488 | Illumina | United States | 100 | 4044579555 |
| PRJNA255922 | | periodontitis | SRR1779492 | Illumina | United States | 100 | 2359086524 |
| PRJNA255922 | | periodontitis | SRR1779495 | Illumina | United States | 100 | 698681158 |
| PRJNA255922 | | SRP | SRR1779497 | Illumina | United States | 100 | 5040986540 |
| PRJNA255922 | | SRP | SRR1779502 | Illumina | United States | 100 | 5769488561 |
| PRJNA255922 | | SRP | SRR1779506 | Illumina | United States | 100 | 2638377601 |
| PRJNA255922 | | SRP | SRR1779511 | Illumina | United States | 100 | 701844673 |
| PRJNA255922 | | periodontitis | SRR1779378 | Illumina | United States | 100 | 4812714019 |
| PRJNA255922 | | SRP | SRR1779403 | Illumina | United States | 100 | 6808471092 |
| PRJNA255922 | | periodontitis | SRR1783028 | Illumina | United States | 100 | 4075499243 |
| PRJNA255922 | | periodontitis | SRR1783032 | Illumina | United States | 100 | 2228264566 |
| PRJNA255922 | | SRP | SRR1783079 | Illumina | United States | 100 | 5722600905 |
| PRJNA255922 | | SRP | SRR1783718 | Illumina | United States | 100 | 3168303798 |
| PRJNA255922 | | periodontitis | SRR1783722 | Illumina | United States | 100 | 1734961396 |
| PRJNA255922 | | periodontitis | SRR1783724 | Illumina | United States | 100 | 4521811885 |
| PRJNA255922 | | SRP | SRR1783757 | Illumina | United States | 100 | 4991376068 |
| PRJNA255922 | | SRP | SRR1783758 | Illumina | United States | 100 | 7328272053 |
| PRJNA255922 | | periodontitis | SRR1794083 | Illumina | United States | 100 | 9271763470 |
| PRJNA255922 | | periodontitis | SRR1794618 | Illumina | United States | 100 | 8794207128 |
| PRJNA255922 | | SRP | SRR1794621 | Illumina | United States | 100 | 1065070031 |
| PRJNA255922 | | SRP | SRR1794623 | Illumina | United States | 100 | 3218966329 |
| PRJNA255922 | | periodontitis | SRR1783760 | Illumina | United States | 100 | 2597362884 |
| PRJNA255922 | | periodontitis | SRR1783763 | Illumina | United States | 100 | 1521676706 |
| PRJNA255922 | | SRP | SRR1783767 | Illumina | United States | 100 | 6834644754 |
| PRJNA255922 | | SRP | SRR1783768 | Illumina | United States | 100 | 1309530473 |
| PRJNA255922 | | periodontitis | SRR1783769 | Illumina | United States | 100 | 4642599535 |
| PRJNA255922 | | periodontitis | SRR1783771 | Illumina | United States | 100 | 2281883760 |
| PRJNA255922 | | SRP | SRR1783774 | Illumina | United States | 100 | 6180443966 |
| PRJNA255922 | | SRP | SRR1783775 | Illumina | United States | 100 | 4271526474 |
| PRJNA255922 | | periodontitis | SRR1794794 | Illumina | United States | 100 | 1945357329 |
| PRJNA255922 | | periodontitis | SRR1795007 | Illumina | United States | 100 | 6232563854 |
| PRJNA255922 | | SRP | SRR1795008 | Illumina | United States | 100 | 5595029746 |
| PRJNA255922 | | SRP | SRR1795009 | Illumina | United States | 100 | 6848098169 |
| PRJNA255922 | | periodontitis | SRR1795012 | Illumina | United States | 100 | 1606644861 |
| PRJNA255922 | | periodontitis | SRR1795016 | Illumina | United States | 100 | 3348757491 |
| PRJNA255922 | | SRP | SRR1795017 | Illumina | United States | 100 | 2418074450 |
| PRJNA255922 | | SRP | SRR1795018 | Illumina | United States | 100 | 6917270433 |
| PRJNA255922 | | periodontitis | SRR1795019 | Illumina | United States | 100 | 5335468831 |
| PRJNA255922 | | periodontitis | SRR1795020 | Illumina | United States | 100 | 3955813069 |
| PRJNA255922 | | SRP | SRR1795229 | Illumina | United States | 100 | 6333533695 |
| PRJNA255922 | | SRP | SRR1795230 | Illumina | United States | 100 | 6313169406 |
| PRJNA528558 | | periodontitis | SRR8786260 | Illumina | China | 100 | 4623786000 |
| PRJNA528558 | | periodontitis | SRR8786261 | Illumina | China | 100 | 4636940100 |
| PRJNA528558 | | periodontitis | SRR8786262 | Illumina | China | 100 | 4595020500 |
| PRJNA528558 | | periodontitis | SRR8786263 | Illumina | China | 100 | 4498272900 |
| PRJNA528558 | | health | SRR8786264 | Illumina | China | 100 | 4625246700 |
| PRJNA528558 | | health | SRR8786265 | Illumina | China | 100 | 4561149300 |
| PRJNA528558 | | periodontitis | SRR8786266 | Illumina | China | 100 | 4312986900 |
| PRJNA528558 | | health | SRR8786267 | Illumina | China | 100 | 4612334400 |
| PRJNA528558 | | periodontitis | SRR8786268 | Illumina | China | 100 | 4467379500 |
| PRJNA625082 | | health | SRR11562254 | Illumina | United States | 150 | 5586770141 |
| PRJNA625082 | | health | SRR11562255 | Illumina | United States | 150 | 8613591525 |
| PRJNA625082 | | health | SRR11562257 | Illumina | United States | 150 | 2370146058 |
| PRJNA625082 | | health | SRR11562268 | Illumina | United States | 150 | 8767113414 |
| PRJNA625082 | | health | SRR11562279 | Illumina | United States | 150 | 6112088737 |
| PRJNA625082 | | health | SRR11562290 | Illumina | United States | 150 | 6745879401 |
| PRJNA625082 | | health | SRR11562296 | Illumina | United States | 150 | 6239914521 |
| PRJNA625082 | | health | SRR11562297 | Illumina | United States | 150 | 6002735280 |
| PRJNA625082 | | health | SRR11562298 | Illumina | United States | 150 | 2251913506 |
| PRJNA625082 | | health | SRR11562299 | Illumina | United States | 150 | 2630721990 |
| PRJNA625082 | | health | SRR11562300 | Illumina | United States | 150 | 10466520437 |
| PRJNA625082 | | health | SRR11562301 | Illumina | United States | 150 | 8995027711 |
| PRJNA625082 | | health | SRR11562302 | Illumina | United States | 150 | 7983845198 |
| PRJNA625082 | | health | SRR11562303 | Illumina | United States | 150 | 6263367617 |
| PRJNA625082 | | health | SRR11562304 | Illumina | United States | 150 | 15824342893 |
| PRJNA625082 | | health | SRR11562305 | Illumina | United States | 150 | 7749799162 |
| PRJNA625082 | | health | SRR11562306 | Illumina | United States | 150 | 7506100369 |
| PRJNA625082 | | health | SRR11562307 | Illumina | United States | 150 | 6221234359 |
| PRJNA625082 | | health | SRR11562308 | Illumina | United States | 150 | 8877438561 |
| PRJNA625082 | | health | SRR11562309 | Illumina | United States | 150 | 7252559582 |
| PRJNA625082 | | health | SRR11562310 | Illumina | United States | 150 | 8145057632 |
| PRJNA625082 | | health | SRR11562311 | Illumina | United States | 150 | 7476714335 |
| PRJNA625082 | | health | SRR11562312 | Illumina | United States | 150 | 8139060392 |
| PRJNA625082 | | health | SRR11562313 | Illumina | United States | 150 | 7233965638 |
| PRJNA625082 | | health | SRR11562314 | Illumina | United States | 150 | 7558372289 |
| PRJNA625082 | | health | SRR11562315 | Illumina | United States | 150 | 1688250043 |
| PRJNA625082 | | health | SRR11562316 | Illumina | United States | 150 | 5548405755 |
| PRJNA625082 | | health | SRR11562317 | Illumina | United States | 150 | 9258221713 |
| PRJNA625082 | | health | SRR11562318 | Illumina | United States | 150 | 7657334634 |
| PRJNA625082 | | health | SRR11562319 | Illumina | United States | 150 | 7413734688 |
| PRJNA625082 | | health | SRR11562320 | Illumina | United States | 150 | 7346831218 |
| PRJNA625082 | | health | SRR11562321 | Illumina | United States | 150 | 6554836149 |
| PRJNA625082 | | health | SRR11562322 | Illumina | United States | 150 | 10509016916 |
| PRJNA625082 | | health | SRR11562323 | Illumina | United States | 150 | 8970313687 |
| PRJNA625082 | | health | SRR11562324 | Illumina | United States | 150 | 5604195738 |
| PRJNA230363 | | health | SRR1044006 | Illumina | China | 101 | 1851933000 |
| PRJNA230363 | | periodontitis | SRR2034640 | Illumina | China | 101 | 1527597600 |
| PRJNA230363 | | periodontitis | SRR2034639 | Illumina | China | 101 | 1826518800 |
| PRJNA230363 | | periodontitis | SRR2034638 | Illumina | China | 101 | 1156981200 |
| PRJNA230363 | | periodontitis | SRR2034637 | Illumina | China | 101 | 807300400 |
| PRJNA230363 | | health | SRR1045100 | Illumina | China | 101 | 327077800 |
| PRJNA230363 | | health | SRR1045099 | Illumina | China | 101 | 760560200 |
| PRJNA230363 | | health | SRR1045098 | Illumina | China | 101 | 469959400 |
| PRJNA230363 | | periodontitis | SRR1045097 | Illumina | China | 101 | 1520026000 |
| PRJNA230363 | | periodontitis | SRR1045096 | Illumina | China | 101 | 1193169800 |
| PRJNA230363 | | periodontitis | SRR1045095 | Illumina | China | 101 | 812243400 |
| PRJNA230363 | | periodontitis | SRR1045094 | Illumina | China | 101 | 889255600 |
| PRJNA230363 | | periodontitis | SRR1045093 | Illumina | China | 101 | 1057460600 |
| PRJNA230363 | | periodontitis | SRR1045092 | Illumina | China | 101 | 979296000 |
| PRJNA230363 | | health | SRR1044036 | Illumina | China | 101 | 529395600 |
| PRJNA230363 | | health | SRR1044035 | Illumina | China | 101 | 1232079400 |
| PRJNA230363 | | health | SRR1044034 | Illumina | China | 101 | 1330775200 |
| PRJNA230363 | | health | SRR1044033 | Illumina | China | 101 | 1184173600 |
| PRJNA230363 | | health | SRR1044032 | Illumina | China | 101 | 831392600 |
| PRJNA230363 | | health | SRR1044017 | Illumina | China | 101 | 760029800 |
